# Novel water-soluble lignin derivative BP-Cx-1: identification of components and screening of potential targets *in silico* and *in vitro*

**DOI:** 10.18632/oncotarget.24990

**Published:** 2018-04-06

**Authors:** Elena I. Fedoros, Alexey A. Orlov, Alexander Zherebker, Ekaterina A. Gubareva, Mikhail A. Maydin, Andrey I. Konstantinov, Konstantin A. Krasnov, Ruben N. Karapetian, Ekaterina I. Izotova, Sergey E. Pigarev, Andrey V. Panchenko, Margarita L. Tyndyk, Dmitry I. Osolodkin, Evgeny N. Nikolaev, Irina V. Perminova, Vladimir N. Anisimov

**Affiliations:** ^1^ N.N. Petrov National Medical Research Center of Oncology, Saint-Petersburg 197758, Russia; ^2^ Department of Chemistry, Lomonosov Moscow State University, Moscow 119991, Russia; ^3^ Institute of Toxicology, Federal Medical-Biological Agency, Saint-Petersburg 192019, Russia; ^4^ CHEMDIV LTD, Moscow District, Khimki 141400, Russia; ^5^ Nobel LTD, Saint-Petersburg 192012, Russia; ^6^ Institute of Poliomyelitis and Viral Encephalitides, Chumakov FSC R&D IBP RAS, Moscow 108819, Russia; ^7^ Sechenov First Moscow State Medical University, Moscow 119991, Russia; ^8^ Skolkovo Institute of Science and Technology, Skolkovo 143025, Russia; ^9^ Institute for Energy Problems of Chemical Physics, Russian Academy of Sciences, Moscow 119334, Russia; ^10^ Orekhovich Institute of Biomedical Chemistry, Russian Academy of Medical Sciences, Moscow 119121, Russia

**Keywords:** cancer, polyphenols, molecular targets, BP-Cx-1, ChEMBL

## Abstract

Identification of molecular targets and mechanism of action is always a challenge, in particular – for natural compounds due to inherent chemical complexity. BP-Cx-1 is a water-soluble modification of hydrolyzed lignin used as the platform for a portfolio of innovative pharmacological products aimed for therapy and supportive care of oncological patients. The present study describes a new approach, which combines *in vitro* screening of potential molecular targets for BP-Cx-1 using Diversity Profile - P9 panel by Eurofins Cerep (France) with a search of possible active components *in silico* in ChEMBL - manually curated chemical database of bioactive molecules with drug-like properties. The results of diversity assay demonstrate that BP-Cx-1 has multiple biological effects on neurotransmitters receptors, ligand-gated ion channels and transporters. Of particular importance is that the major part of identified molecular targets are involved in modulation of inflammation and immune response and might be related to tumorigenesis. Characterization of molecular composition of BP-Cx-1 with Fourier Transform Ion Cyclotron Resonance Mass Spectrometry and subsequent identification of possible active components by searching for molecular matches *in silico* in ChEMBL indicated polyphenolic components, nominally, flavonoids, sapogenins, phenanthrenes, as the major carriers of biological activity of BP-Cx-1. *In vitro* and *in silico* target screening yielded overlapping lists of proteins: adenosine receptors, dopamine receptor DRD4, glucocorticoid receptor, serotonin receptor 5-HT1, prostaglandin receptors, muscarinic cholinergic receptor, GABAA receptor. The pleiotropic molecular activities of polyphenolic components are beneficial in treatment of multifactorial disorders such as diseases associated with chronic inflammation and cancer.

## INTRODUCTION

Lignin and its derivatives are considered as good candidates for development of novel medicinal products thanks to their low toxicity and broad range of biological activity. Lignin derivatives are known to exhibit antiviral [1, 2], anti-inflammatory [3], antidiabetic [4, 5] and antitumor [6, 7] effects. In addition, hydrolyzed lignin is used as enterosorbent [8], as well as for production of biodegradable and safe drug-delivery nanoparticles, e.g., antibacterial silver-compositions [9, 10]. Antioxidant activity, which is inherent to polyphenolic compounds, is believed to be the leading mode of action of lignin-like compounds [11, 12, 13].

Due to poor solubility in water, lignin and its derivatives demonstrate low bioavailability in animals. Hence, preparation of water-soluble modifications of lignin, which are most suitable for development of pharmacologically active substances, is of particular interest. Liquid-phase hydrolysis is the method of choice for preparing water-soluble lignin derivatives. In our previous studies, we used a lignin-based enterosorbent - Polyphepan^®^ as the starting material and treated it in the alkaline medium at the elevated pressure and temperature with continuous supply of oxygen, to form the target water-soluble modification of lignin – BP-Cx-1 [14]. In line with the protocol of its production, BP-Cx-1 is a multicomponent composition of polyphenolic compounds. It was further used as a ligand for preparation of novel pharmaceutical products BP-C1, BP-C2, and BP-C3.

Platinum containing antineoplastic investigational medicinal product (IMP) BP-C1 is at the moment on the stage of clinical trials in patients with metastatic breast cancer [15]. Investigational medicinal product BP-C2 is a composition of BP-Cx-1 with ammonium molybdate. It exposes radioprotective effects through protection and reconstruction of hematopoiesis in irradiated rats [16], inhibits radiation-induced skin damage in mice [17], and improves quality of life of cancer patients undergoing chemotherapy [18]. BP-C3 is a composition of BP-Cx-1 with iron complex, selenium, ascorbic acid, and retinol. It exhibits geroprotective activity [19] and reduces 5-fluorouracil toxicity in mice by accelerating recovery of leukopoiesis and protecting small intestine epithelium [20].

Despite multiple studies published on biological effects of lignin derivatives, molecular mechanism of their action remains unknown. This is because application of existing characterization methods to multicomponent compositions of lignin and its derivatives is very challenging. Fourier transform ion cyclotron resonance mass-spectrometry (FTICR MS) with mild ionization techniques is the only method, which is capable to resolve this issue. Due to its high resolution and mass accuracy, this method detects thousands of molecular peaks in complex mixtures without preliminary fractionation [21]. The power of this method enables exact assignment of thousands formulae, which could be used to evaluate the pathways of product formation as well as to suggest individual components responsible for bioactivity. Recently, FTICR MS was applied to explore homologues series in lignin derivatives [22]. Despite clear advantage of FTICR MS over other analytical techniques, the limitation of this method is a tolerance to isomers. The possible way to overcome this limitation is the application of selective chemical modification with enumeration of reactive centers [23]. Searching for well-known molecules with matching molecular masses and the described biological activity profiles is another feasible approach.

The identification of the molecular targets for bioactive compounds is an essential step in every drug discovery project [24]. In order to complement *in vitro* experiments numerous *in silico* approaches based on either information about the structures of biotargets or ligands were developed [25, 26]. These techniques were broadly applied in natural compound drug research [27, 28]. However, in case of compound mixtures, when structural information is limited and the individual compounds are hard to elucidate, a range of strategies, united under a term ‘dereplication’, were suggested [29]. Dereplication, in a broad sense, can be defined as a process of quickly identifying known chemotypes [29]. Different variants of dereplication strategies were successfully applied for chemical profiling of natural products by means of combining NMR, MS data and search in compound and bioactivity databases [29].

The present study describes a dereplication strategy, combining FTICR MS data, *in vitro* screening of potential molecular targets for water-soluble lignin-based ligand BP-Cx-1 using Diversity Profile - P9 panel by Eurofins Cerep (France), with identification of possible active components of BP-Cx-1 by means of searching for their molecular formulas in silico in ChEMBL, which is large scale bioactive compounds database.

## RESULTS AND DISCUSSION

### BP-Cx-1 characterization

The BP-Cx-1 was characterized using a number of methods. Elemental composition was determined using automatic elemental analysis. The content of carbon was 66.7%, hydrogen–4.82%, and oxygen–28.48%, which corresponded to H/C ratio of 0.87 and O/C ratio of 0.32. The obtained data demonstrate low oxidation degree and high contribution of aromatic fragments into molecular structures of the water-soluble modification of lignin – BP-Cx-1.

The structural group composition was determined using the most powerful method of structural analysis - NMR spectroscopy. ^13^C and ^1^H NMR spectra are shown in Supplementary Figures 1 and 2. The peaks at 55–58 ppm in ^13^C NMR spectrum and at 3.3–4.3 ppm in ^1^H NMR spectrum are related to methoxy groups which are constitutive parts of lignin (Supplementary Figure 3). High values of spectral density in the range from 108 to 165 ppm of ^13^C NMR and in the range from 6.2 to 7.9 ppm of ^1^H NMR are consistent with predominantly aromatic nature of the product used in this study. Quantitative data of ^13^C and ^1^H NMR spectroscopy are given in Table 1. They indicate that aromatic compounds represent main part of BP-Cx-1. It also contains carboxyl and carbonyl groups, which are formed during oxidation of the starting lignin material.

**Table 1:** Structural group composition of BP-Cx-1 as measured by ^13^C and ^1^H NMR spectroscopy

Carbon content in the structural fragments,%						
O = CRR’	COO	C_Ar-O(H,R)_	C_Ar_	C_Alk-O_	OCH_3_	C_Alk_
4	6	8	45	15	7	15
**Hydrogen content in the structural fragments,%**						
	COOH+ArOH		ArH		CH_3_O	AlkH
	6.8		36		34.5	19.7

In general, the structural group composition of the product under study agrees well with the data for the lignin starting material - a cross-linked polymer composed of phenylpropanoid units [30].

### High-resolution mass-spectrometry

**Figure 1:**
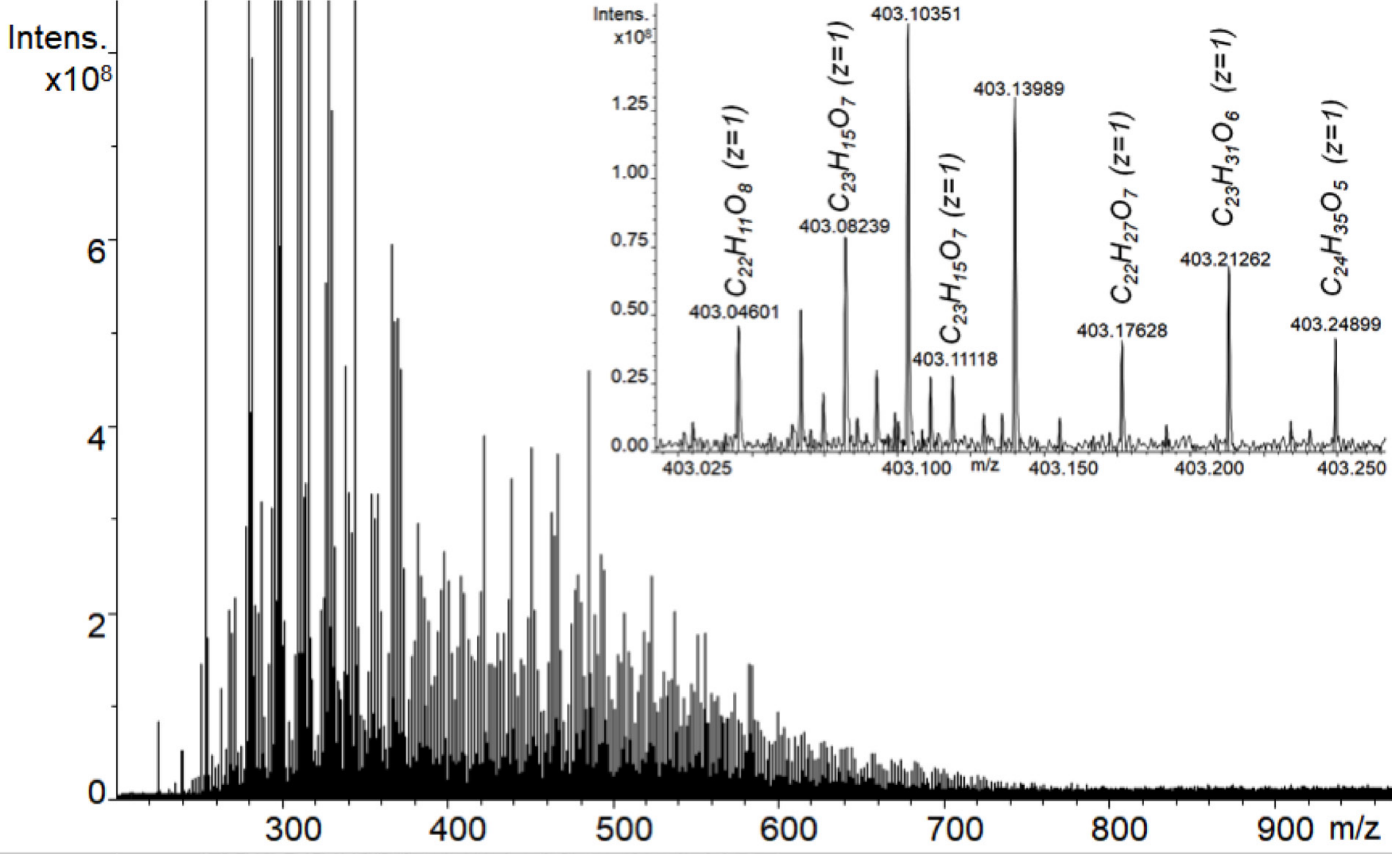
ESI FTICR mass-spectrum of the lignin derivative used in this study - BP-Cx-1 Insert shows the presence of singly and doubly charged ions within 0.25 Da of mass-spectrum fragment.

FTICR mass-spectrum is presented in Figure 1. The most intensive peaks in the mass-spectrum correspond to singly charged ions within 200-900 m/z window. In addition, low-intensity peaks of doubly charged ions could be detected (shown in the inset in Figure 1), which extend mass window up to 1600 Da. High mass accuracy of the FTICR MS enabled identification of exact CHO molecular formulas for 2146 singly- and 1602 doubly-charged ions.

The FTICR MS data were used to calculate number-averaged elemental composition of the sample used in this study, which accounted for 65.7% (C), 5.52 (H), and 28.77 (O). The obtained content of elements yielded H/C ratio of 1.01 and O/C ratio of 0.33. The MS-derived values of H content and the H/C ratio are somewhat higher as compared to those measured by the elemental analysis, which might be indicative of preferential ionization of hydrogen enriched aliphatic acids as compared to phenolic compartments of the BP-Cx-1 sample [31]. Still, the data on elemental composition of the BP-Cx1 sample obtained by the two methods – ESI FTICR MS and elemental analysis – are rather consistent and indicative of polyphenolic nature of the sample under study.

**Figure 2:**
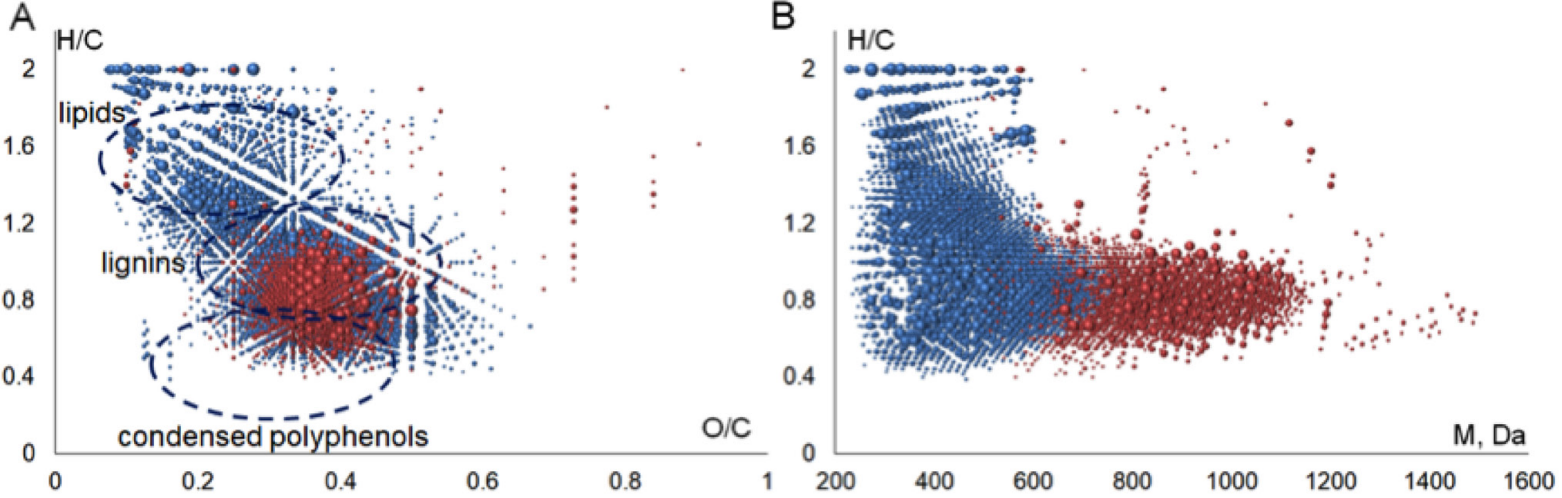
Visualization of FTICR MS data obtained for the BP-Cx-1 lignin derivative: (**A**) Van Krevelen diagram, (**B**) H/C versus molecular weight diagram. The molecular compositions of singly and doubly charged ions are highlighted in blue and red colors, respectively.

The molecular formulae assigned to FTICR MS data were plotted in Van Krevelen diagram, which is widely used for visualization of molecular composition of the complex matrices [32]. It represents a 2D plot of H/C versus O/C ratios constructed from all the determined molecular formulae (Figure 2A). The advantage of this diagram is that it demonstrates the full elemental composition space occupied by the sample rather than the average bulk values. The obtained Van Krevelen diagram shows that the singly-charged ions (highlighted in blue color) contribute the most into molecular heterogeneity of the BP-Cx-1 sample–the corresponding dots are scattered all over the diagram field, whereas the molecular compositions of doubly-charged ions are much more concentrated (highlighted in red color) and located mostly within a narrow region of H/C values from 0.5 to 1 and O/C values from 0.3 to 0.5.

The convenient approach to describing MS-derived data in Van Krevelen diagram is an assignment of identified compounds to different molecular classes: lignin, tannins, lipids, etc, as it is reported in [33]. This enabled us for assigning the majority of doubly-charged ions to lignin-like components because they are located in the region, which is typical for phenylrpopanoic structures (Figure 2A), whereas the molecular formulae assigned to singly-charged ions occupy the regions, which are typical for lipids, lignins and condensed aromatics. The significant differences between elemental compositions of singly and doubly-charged ions are also revealed by closer consideration of molecular mass distribution of the identified ions, which is shown in Figure 2B.

So, H/C versus molecular mass plot shows that all medium-molecular weight compounds, which exceed 600 Da, correspond to lignin-like molecules. At the same time, saturated (aliphatic) and condensed species are characterized by lower molecular weight. This might be indicative of depolymerization of the starting lignin material, which gives rise to formation of heterogenic products. Aromatic ring cleavage and condensation of phenols are among the most probable reactions: the former might result in formation of aliphatic molecules with H/C>1.2, whereas the latter might lead to formation of condensed products with H/C<0.5 (Figure 2B). In the case of BP-Cx-1, both reactions might be induced by an attack of hydroxyl radicals present in alkaline conditions onto the polyphenolic structures of the starting lignin material, as it was described, e.g., in [34]. We also cannot exclude the presence of extractives (terpenoids) in the starting lignin material, which could contribute to the saturated low molecular weight components.

The conducted studies allowed us to conclude that the BP-Cx-1, lignin derivative used in this study, is characterized by dominating contribution of polyphenolic compounds with molecular weights ranging from 300 to 1200 Da along with the presence of low molecular weight compartments of aliphatic character (e.g., terpenoids, fatty acids), and condensed aromatic compounds (e.g., flavonoids).

### *In vitro* studies

Given the results of structural characterization of the lignin derivative under study, which revealed its molecular diversity, we have started exploration of its potential action targets, using the Diversity profile panel of Eurofins, which is composed of 71 binding and 27 enzyme assays (see Supplementary File 1). The binding assay panel has roughly an equal number of selective, central and peripheral therapeutically relevant targets. The enzyme assay panel is particularly focused on phosphatases and specific enzymes involved in cell cycle regulation. This profile is designed for the assessment of the biological diversity and potential adverse activity of Lead discovery candidates. Only the results of the binding assays were used in the present work.

The corresponding assay of BP-Cx-1 biological activity was conducted at the highest achievable concentration of 0.0042% (V/V). The thirteen cases of nonspecific interference of BP-Cx-1 with the assay and the pronounced BP-Cx-1 binding (over 50%) to 22 targets (Table 2) were identified.

**Table 2:** Binding targets of BP-Cx-1 identified by Diversity Profile–P9 panel

Receptor family	Target	Protein/protein family symbol	Inhibition%
Adenosine receptors	A1 (h)	ADORA1	62.1%
	A2A (h)	ADORA2A	86.6%
Adrenergic receptors	α2	ADRA2A-C	52.2%
	β1 (h)	ADRB1	51.5%
	β2 (h)	ADRB2	71.4%
GABAA (γ-Aminobutyric acid)	BZD	GABRA1	53.8%
Glutamate	AMPA	GRIA 1-4	90.9%
	Kainate	GRIK 1-5	84.3%
Bradykinin receptors	B1 (h)	BDKRB1	76.7%
Dopamine receptors	D4.4 (h)	DRD4	62.7%
Histamine receptors	H1 (h)	HRH1	57.6%
Cholinergic receptors	α4β2 (h)	CHRNB2, CHRNA4	73.5%
	M	CHRM1-5	96.3%
Vasopressin receptor	V2 (h)	AVPR2	69.8%
	V1a (h)	AVPR1A	56.4%
Ion channels	KATP	KCNJ	50.3%
Transporters	GABA	SLC6A1	71.5%
Thyroid Stimulating Hormone receptor	TRH1 (h)	TSHR	68.8%
Glucocorticoid receptor	GR (h)	NR3C1	63.2%
Serotonin receptor	5-HT1	HTR1 A,B,D,E,F	81%
Purinergic receptors	P2X	P2RX1-7	64%
	P2Y	P2RY1,2,4,6,11–14	52.6%
Prostanoid receptors	EP2 (h)	PTGER2	52.4%
	IP (PGI2)	PTGIR	68.7%

The results show that interaction of BP-Cx-1 with multiple targets was detected. The majority of the identified targets belong to neurotransmitters receptors, ligand-gated ion channels and transporters, which are expressed in central and peripheral nervous systems. Given that drugs, which contain BP-Cx-1 ligand, are developed for antitumor therapy or as supportive care for cancer patients [35], of particular interest was to identify molecular targets, which are involved in modulation of inflammation and immune response.

Adenosine, adrenergic, bradykinin receptors, glucocorticoid receptor, histamine receptors, acetylcholine muscarinic receptors, purinergic receptors and prostanoid receptors are known to be involved in regulation of inflammation response [36]. Inhibition of bradykinin receptor B1 [37, 38], muscarinic receptors M1 and M2 [39], histamine receptor H1 [40] and prostanoid receptors EP2 [41], as well as activation of glucocorticoid receptor GR [42] and adenosine receptors A(1), A(2A) [43, 44], is beneficial for reducing inflammation and pain.

There is reported evidence suggesting that chronic inflammations are tightly associated with cancer [45, 36]. This might indicate specific role of inflammation promoting receptors in tumorigenesis. The tumors recruit multiple paracrine, autocrine and systemic signaling factors for maintenance of high proliferative and metabolic level and for avoiding host immune response. As a result, simultaneous targeting of several proteins may be beneficial for treatment of the cancer patients.

Inhibitors and agonists of many receptors are currently considered as antitumor drug candidates. Thus, glutamate bradykinin and AMPA receptors have been implicated in cancer migration, invasion and metastasis [46, 47, 48]. Some data on prostaglandin receptor EP2 indicate that it is highly expressed in a variety of cancer cells and exacerbates disease pathology [49, 50, 41, 51]. Adenosine A(1) and A(2A) receptors agonists exhibit immunosuppressive effect in tumor stroma [52, 53]; this effect can be amplified by co-administration of selective inhibitors of purinergic receptors [54]. Dopamine D4 receptor inhibitors, which affect autophagy in glioblastoma stem cells and induce apoptosis were also demonstrated to have antitumor activity [55]. This leads to suggestions that β-adrenoceptors might be valuable drug targets to control the deleterious effects of β-adrenergic system in tumor development along with psychological stress in cancer patients [56, 57].

The direct experimental evidence with regard to possible effect of BP-Cx-1 onto various receptors, taking part in inflammatory reactions and tumor development, as well as with regard to its pharmacologically active concentrations is missing. However, the data from clinical trials of BP-Cx-1 containing products, as well as laboratory animal testing allows us a suggestion that its numerously documented cumulative anti-inflammatory and antitumor effect in cancer patients may in part be attributed to the direct ligand-receptor interaction.

### ChEMBL data mining

For suggesting structures, which might be related to the BP-Cx-1 bioactivity profile, we used the FTICRMS data on molecular formulae, which were identified in this sample (3748 formulas). We used these formulas for manual search in ChEMBL database. The related structures were extracted along with their bioactivity data. The text search was conducted in the target_dictionary.pref_name and assays.description fields, which contain information about protein target and biological assay conditions. This enabled extraction of 1474 relevant assays corresponding to 1316 ChEMBL compounds with interpretable activity values. Due to diversity of ChEMBL assays and activity types, the activity values were hashed to boolean flags, where ‘1’ and ‘0’ denoted the presence or absence of the compound activity against the target. Individual flags were summarized in boolean fingerprints (Supplementary File 2, Figure 3), which represent compound bioactivity profiles against all the targets screened. The obtained bioactivity profiles were compared with the experimental data by constructing the so-called “fingerprints”. We proposed a parameter of the fitness of a ChEMBL compound to the BP-Cx-1 bioactivity profile: values of ‘1’ and ‘–1’ corresponded to the match or mismatch of activity of the ChEMBL compound and BP-Cx-1 against the target, respectively, and ‘0’ was assigned in the absence of data (Supplementary File 3, Figure 3). All values were summed up giving fit_score - the final value, which characterized the fitness of a compound to the BP-Cx-1 bioactivity profile. Thus, 56 would represent the fit_score for an ideally fitted compound, that was tested and demonstrated matching activity in all 56 receptors. The maximum fit_score found for the ChEMBL compounds was 4. The fit_score ≥1 (well-fitted compounds) was obtained for 354 ChEMBL compounds.

**Figure 3:**
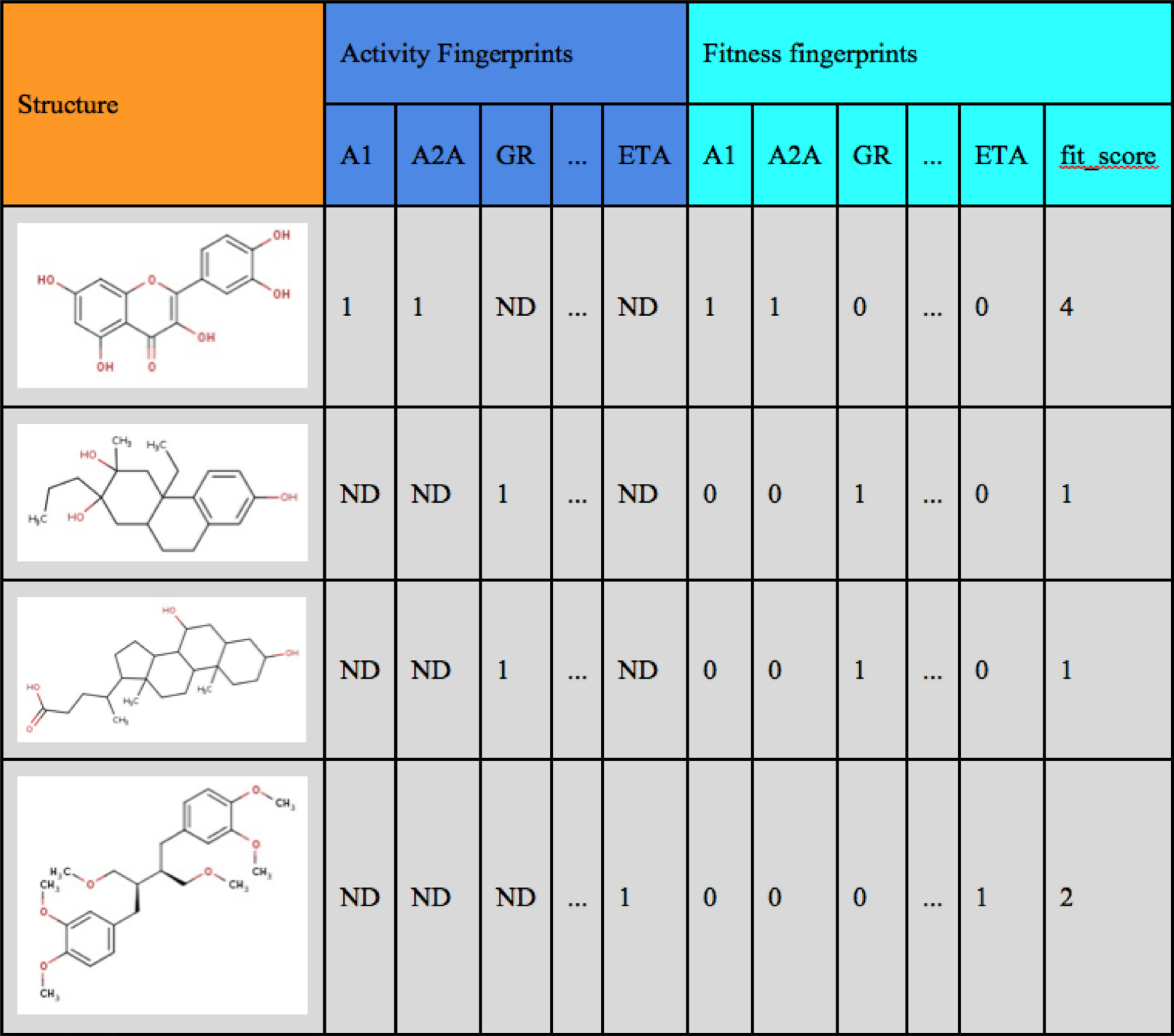
Examples of Boolean bioactivity fingerprints

**Figure 4:**
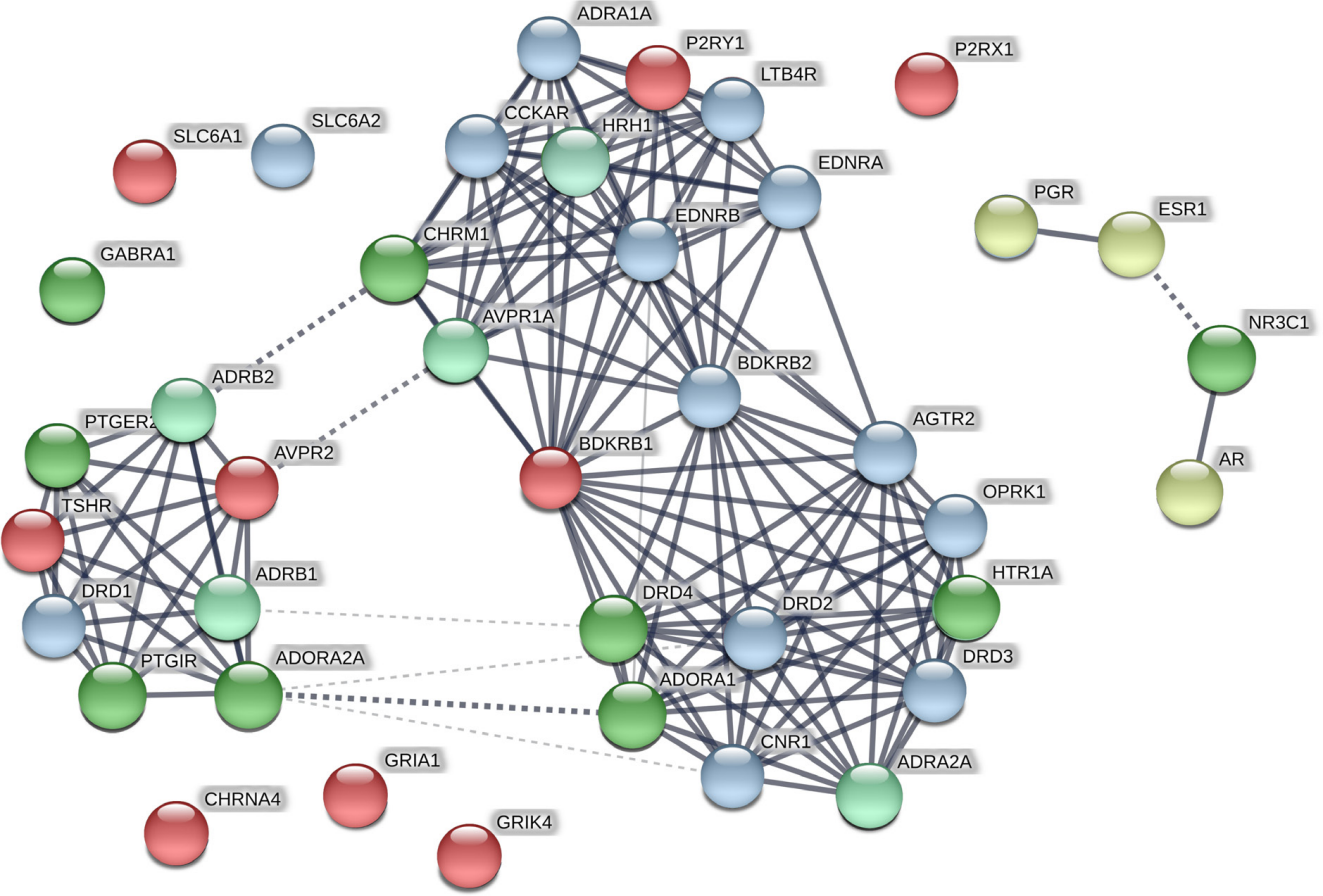
STRING protein association network for BP-Cx-1 and ChEMBL compounds targets Bright-green–targets: activity of the well-fitted ChEMBL compound matched activity of BP-Cx-1; Light-green–targets: activity of other ChEMBL compounds matched activity of BP-Cx-1; Yellow–targets: activity of the well-fitted ChEMBL compound did not match activity of BP-Cx-1 (binding by BP-Cx-1 below 50%); Blue–targets: activity of other ChEMBL compounds did not match activity of BP-Cx-1 (binding by BP-Cx-1 below 50%); Red–BP-Cx-1 targets: no matching activity records were identified in the ChEMBL database.

Of the 1316 ChEMBL compounds, 1005 had the activity against various targets matching that of BP-Cx-1, and 1968 did not match activity of BP-Cx-1. For the 354 well-fitted ChEMBL compounds these values were 396 and 6, correspondingly (Supplementary File 3). Data on activity of the ChEMBL compounds and BP-Cx-1 against the Diversity Profile targets (receptors and transporters) are graphically presented as a STRING protein association network in Figure 4. Network approach was used to analyze functional clusters of BP-Cx1 targets established *in vitro* and additional probable targets identified in silico.

As intracellular signaling resembles network rather than straightforward pathways, targeting several connected proteins would influence the pathway more efficiently than interaction with a single target. Network analysis in STRING revealed that the targets form a network which is enriched in interactions, i.e. it has more interactions among proteins than a random set of proteins (enrichment *p*-value: < 1.0e–16). Interaction enrichment implies that proteins are at least partly connected and regulate the same function. In our network (Figure 4), some biological processes according to GO (gene ontology database) were enriched, including G-protein coupled receptor signaling, blood circulation, cellular calcium ion homeostasis, regulation of cell proliferation, inflammatory response.

As can be seen from Figure 4, established activity of ChEMBL compounds was matching that of BP-Cx-1 for 14 out of the 22 targets listed in Table 2, at that, well-fitted ChEMBL compounds had the activity against 9 targets (Supplementary File 3). The highest number of ChEMBL compounds with activities matching those of BP-Cx-1 were found for the following BP-Cx-1 targets: adenosine receptors A1 (ADORA1) – 71 records of matching activity among 91 tested compounds and 20 out of 20 records for the well-fitted ChEMBL compounds, A2 (ADORA2A) – 52 active out of the 63 tested (7 out of 7 for the well-fitted ChEMBL compounds), dopamine receptor D4.4 (DRD4) – 65 out of 73 for all ChEMBL compounds and 15 out of 15 for the well-fitted compounds. For GR (NR3C1) glucocorticoid receptor matching activities were identified for 81 out of 103 tested compounds (29 out of 32 for the well-fitted), for 5-HT1 (HTR1 A,B,D,E,F in Table 2, HTR1A in Figure 4) serotonin receptor matching activities were identified for 59 out of 64 tested compounds (6 out of 6 for the well-fitted compounds) and for EP2 (PTGER2) receptor, matching activities were identified for 24 out of 27 compounds and for 10 out of 10 of the well-fitted compounds. A smaller number of matches were identified for I2 (PTRIG) ligands - 8 out of 25 tested compounds with fit_score ≥1 were active (8 out of 8 for the well-fitted compounds) and for the muscarinic (CHRM1-5 in Table 2, CHRM1 in Figure 4) cholinergic receptor – 50 active compounds were identified in ChEMBL (52 records were tested), however, only 1 compound had the fit_score ≥1. Compounds active against BZD site of GABAA receptor (GABRA1) were also identified: 8 compounds were identified, of which 5 were active (2 out of 2 for the well-fitted compounds).

Around 50 activity records against each of adrenergic (α2, ADRA2A; β1, ADRB1; β1, ADRB1) as well as , histamine (H1, HRH1) and vasopressin (V1a, AVPR1A) receptors were identified in ChEMBL database; activity profiles of all corresponding ChEMBL compounds were characterized by the fit_score <1 (Supplementary File 3, Figure 4).

There was a number of receptors in the binding assays of the Diversity Profile–P9 panel, against which activity of BP-Cx-1 was smaller than 50% (Supplementary File 1). ChEMBL compounds were found active against 16 of these receptors (Figure 4). Thus, while 212 out of 283 tested ChEMBL compounds were active against cannabiod (CCKAR) and 145 out of 236 against opiod (OPRK) receptors, BP-Cx-1 had a low affinity to these (9% and 23%, respectively). Likewise, 191 out of 208 and 106 out of 145 ChEMBL compounds, were active against endothelian ETA (ENDRA) and ETB (ENDRB) receptors, respectively.

BP-Cx-1 also demonstrated low affinity to nuclear receptors: 30% for ER (ESR), 13% for PR (PGR) and 36% for AR. 275 out of 353 tested ChEMBL compounds were active against estrogen receptors; 98 out of 108 and 109 out of 136 were active against progesterone and androgen receptors, respectively. Interestingly, single instances of activity against these receptors were identified for the well-fitted ChEMBL compounds. Thus, activity records exist in ChEMBL database for: 4ʹ,5,7-Trihydroxyflavone against estrogen receptor, resorcylic acid lactone with molecular formula C_21_H_28_O_6_ against progesterone receptor and tetracyclic triterpene (C_24_H_30_O_6_) against androgen receptor.

**Figure 5:**
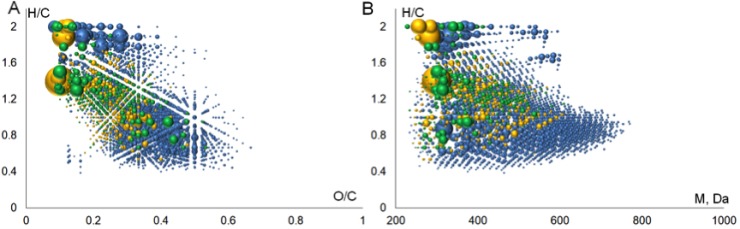
Visualization of derived molecular space (**A**) Van Krevelen and (**B**) H/C versus molecular weight diagram. Blue, orange and green spheres represent formulas for FTICR MS compounds, ChEMBL compounds and ChEMBL well-fitted compounds, respectively.

To compare chemical composition of ChEMBL compounds versus BP-Cx-1 compounds, the molecules found in ChEMBL database were plotted in Van Krevelen diagram and in the H/C vs MW diagram (Figure 5). It can be deduced that the major portion out of 1316 identified formulas corresponded to saturated compounds (Figure 5A) with molecular masses below 500 Da (Figure 5B). By the structural type we assume them to be terpenoids and lignans. For example, the role of lignans in biological activity was reported in our previous work [58]. It should be noted that the database query returned BP-Cx-1 components with the highest relative intensity in mass-spectrum. Given that 354 ChEMBL well-fitted compounds with fit-score ≥1 are low-molecular weight entities with high abundance (Figure 5), we hypothesize that saturated compounds play a significant role in interaction with the identified targets.

Additionally, we calculated Murcko scaffolds for all ChEMBL compounds (Table 3). It can be deduced from Table 3 that the scaffolds of well-fitted compounds match those of furanditerpenes derivatives (Table 3, 1A, 2B), tetracyclic triterpenes (Table 3, sapogenins, 2A, 1B) and their precursors–phenanthrene structures (Table 3, 4A), as well as flavonoids (Table 3, flavanes, 3A) and simple aromatic structures (Table 3, fragments of noncondensed lignin, 5A, 3B). Compounds of these types are part of various natural products [59, 60, 61], and they may also form in the course of successive acidic and alkaline hydrolysis of wood [62].

**Table 3:** Murcko scaffolds distribution for all ChEMBL compounds and ChEMBL compounds with fit_score ≥1 (well fitted to BP-Cx-1 bioactivity)

Well fitted ChEMBL compounds			All ChEMBL compounds		
#	Scaffold	%	#	Scaffold	%
1A	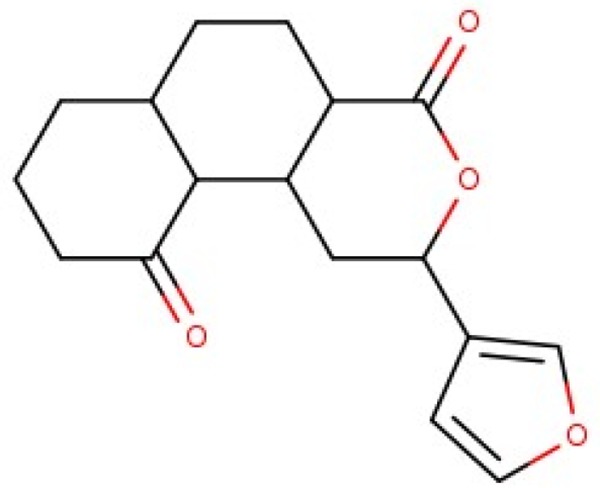	11.1	1B	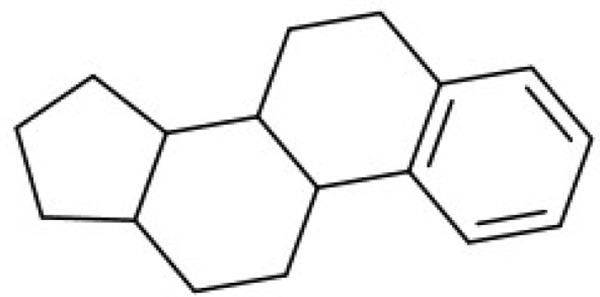	6.3
2A	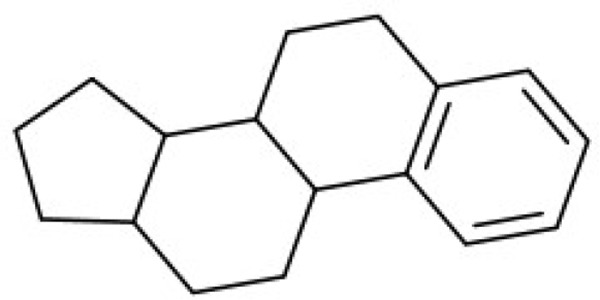	4.5	2B	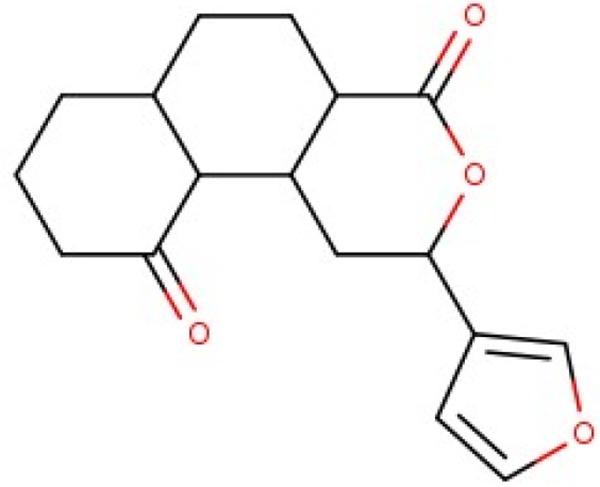	5.6
3A	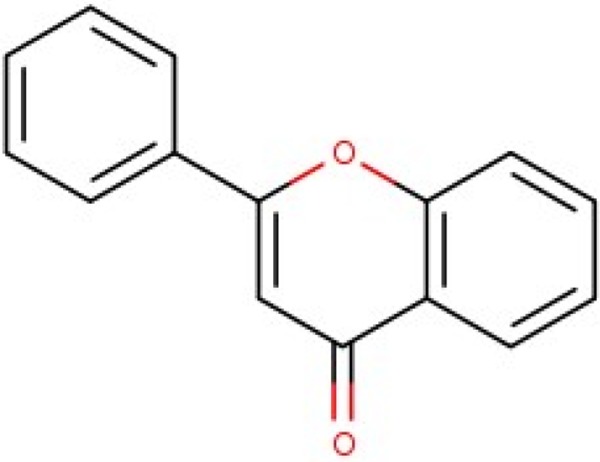	3.7	3B	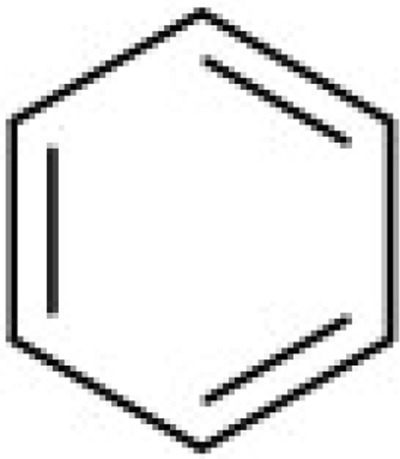	3.3
4A	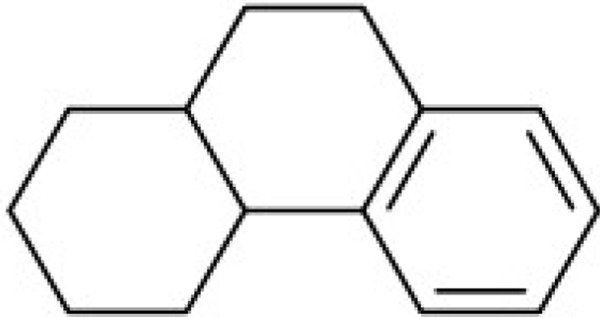	3.1	4B	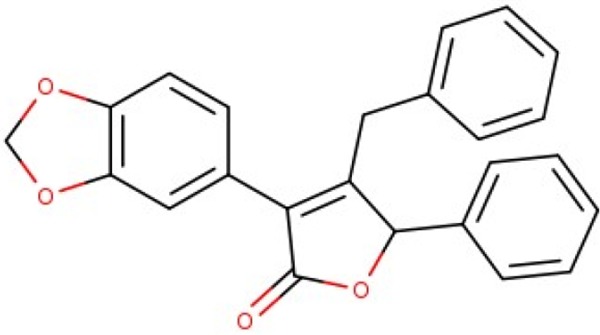	3.0
5A	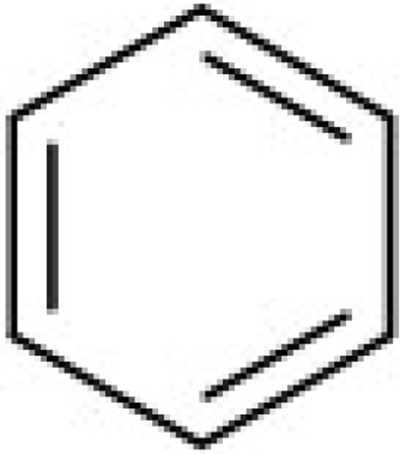	2.8	5B	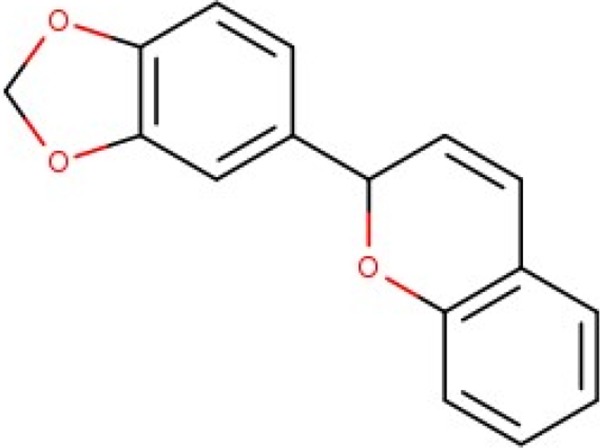	2.2

Polyphenolic compounds of flavonoid nature are the most investigated in the medical field (Table 3, 3A). Antiinflammatory [63], antitumor [64, 65], antiarthritic [66] activities were demonstrated for quercetin. The spectrum of biological activity of genistein isoflavone resembles that of other flavonoids [67, 68]; besides, it is conformationally similar to estradiol and it might act as the latter [69]. It is the estrogen-like activity that is referred to as being responsible for the radioprotective effectiveness of this compound in ARS CD2F1 murine model (DRF 1.16 in case of s.c. prophylactic administration) where it acts via protection of bone marrow progenitor cell populations [70].

It is worthwhile to note that the present study demonstrated moderate activity of the BP-Cx-1 sample towards ER receptors (Supplementary File 1), while in our previous ARS study, efficacy of BP-C2, composition composed of BP-Cx-1 and molybdenum, was similar to that of genistein (DRF = 1.1). The results of that study suggested that the mechanism of action is associated with protection and acceleration of recovery of hematopoietic system of irradiated animals [16]. Apparently, induction of antiinflammatory signaling pathways, though not ER-induced, results in a similar *in vivo* activity of BP-C2.

Given that some flavonoids are reported to traverse the blood-brain barrier, when administered orally and/or parenterally [71], it might be expected that other compounds of this group could act on CNS receptors. The studies dedicated to these effects are very scarce, but in a study of hispidulin, for instance, ability of this compound to act as a ligand in recombinant GABAA/BZD receptors expressed by Xenopus laevis oocytes was demonstrated as well as a pronounced anticonvulsive effect was observed in seizure-prone Mongolian gerbils (Meriones unguiculatus) in a model of epilepsy of oral formulation of this compound at 10 mg/kg body weight [72].

Activity of furane diterpene compounds (Table 3, 1A, 2B) towards CNS receptors such as salvinorin A, a selective high efficacy kappa-opioid receptor (KOPr) agonist, is reported; some intensive studies are performed to evaluate furane diterpene products with a reduced burden of undesirable effects associated with salvinorin A [73].

Earlier reports demonstrated that BP-Cx-1, when administered intravenously, was able to traverse the blood brain barrier [35]. Activity of BP-Cx-1 towards GABAA/BZD receptors and a lack of effect towards opioid receptors (Supplementary File 1) are demonstrated by the results of the present study.

Ginseng, Pulsatilla sp., Aesculus pavia and other plants are the natural sources of triterpene compounds (Table 3, 2A, 1B). Like flavonoids, natural saponines (glycosides) and sapogenins (aglycosides) are characterized by multiple biological activities: antitumor [74–77], antiinflammatory [78], pain reducing [79].

Phenanthrene structures (Table 3, 4A) are mainly obtained from Orchidaceae family plants. Phenanthrenes exhibit various biological activities, among which are anti-inflammatory, antiallergic [80] and cytotoxic [81] effects.

Thus, polyphenols’ “molecular promiscuity”, in a sense that a single molecule has multiple targets, is a characteristic feature for this class of compounds. It is important to note that the targets include diverse protein families such as GPCRs, enzymes, ion channels, transcription factors. Different polyphenolic molecules have been shown to influence signaling pathways associated with inflammation, oxidative stress, cancer, metabolic regulation, etc. [82–84].

## CONCLUSIONS

The reported study, for the first time, employed interdisciplinary approach towards identification of molecular targets and biologically active structures of the novel water-soluble lignin derivative – BP-Cx-1, used as a platform for a portfolio of medicinal products for the main therapy and supportive care of oncological patients.

It was demonstrated that BP-Cx-1 exerts multiple pharmacological effects towards neurotransmitters receptors, ligand-gated ion channels and transporters; most of molecular targets are involved in modulation of inflammation and immune response. Examination of molecular composition of BP-Cx-1 with FTICR MS and subsequent identification of possible active components of BP-Cx-1 by searching for their molecular formulas *in silico* in ChEMBL indicate that the said biological activity may stem from multiple polyphenolic components likely contained in BP-Cx-1: flavonoids, sapogenins, phenanthrenes etc. *In vitro* and in silico target screening yielded not identical, but overlapping lists of proteins. Both methods identified adenosine receptors, dopamine receptor DRD4, glucocorticoid receptor, serotonin receptor 5-HT1, prostaglandin receptors, muscarinic cholinergic receptor, GABAA receptor.

Despite great molecular diversity of polyphenolic compounds and of their targets, biological activities of the known polyphenolic compounds are similar; the most of those activities are the anti-inflammatory and antiproliferative. Even though this class of compounds lacks selectivity towards particular targets, BP-Cx-1 pleiotropic molecular activities may be beneficial in the treatment of multifactorial disorders, such as diseases associated with chronic inflammation (neurodegenerative diseases, rheumatoid arthritis, autoimmune disorders), cancer, type 2 diabetes.

## MATERIALS AND METHODS

### BP-Cx-1 composition

BP-Cx-1 was provided by Nobel Ltd as a powder with 95.9% active substance (batch A1) for the characterization analyses and as a sterile 0.42% ammonia solution (batch X112K14A1) for the *in vitro* studies. In the *in vitro* studies BP-Cx-1 was tested at a concentration of 0.0042% (V/V).

### BP-Cx-1 characterization

Elemental analysis (C, H, and N) was conducted using Vario El Microcube analyzer. Ash content was measured by manual combustion of the test-agent at 850°C. The content of all elements was calculated on ash-free basis. Oxygen concentration was calculated as the difference between a mass of the sample and the ash-corrected amount of CHN.

Quantitative ^13^C solution-state NMR spectra were recorded with Avance NMR spectrometer (Bruker, Germany), operating at 100 MHz carbon-13 frequency. A 50 mg BP-Cx-1 sample was dissolved in 0.6 mL 0.3 N NaOD and transferred into 5 mm NMR tube. ^13^C NMR spectra were acquired with a 5 mm broadband probe, using CPMG pulse sequence with nuclear Overhauser effect suppression by the INVGATE procedure; the acquisition time and relaxation delay were 0.2 s and 7.8 s, respectively. Quantitative distribution of carbon among the main structural fragments of the test-agent was established under these analytical conditions [85]. The assignments were as follows (in ppm): 5–108, aliphatic C atoms (∑C_Alk_) including 50–58, methoxy group (C_CH3O_); 108–165, aromatic C-atoms (∑C_Ar_); 165–187, C atoms of carboxylic and ester groups (COO); 187–220, C atoms of quinonic and keto- groups (C=O).

^1^H NMR spectra were recorded with Bruker AM-400 at 400 MHz, in standard 5.0 mm NMR tubes. Each sample was analyzed in DMSO-d_6_ solution, and then in DMSO-d_6_ with addition of deuterated trifluoroacetic alid 20%(wt). Samples were dissolved at 40 mg/mL. Spectra were recorded at 20°C, pulse delay of 3 s, AT = 1 s, NA = 32. Spectra were analyzed in NUTS (Acorn NMR) software. Five integration regions are considered for this work (in ppm): 1.6–3.3, aliphatic protons (∑H_Alk_); 3.3–4.3, methoxy group protons (∑H_CH3O_), 6.2–7.9 ppm, aromatics and vinylics (∑H_Ar_); 9.4–11.0, protons in aldehyde groups (H_C=O_); 12.6–13.5, protons in carboxylic acid groups.

FTICR mass spectra were acquired with FT MS Bruker Apex Ultra with harmonized cell (Bruker Daltonics) equipped with a 7 T superconducting magnet and electrospray ion source (ESI) in negative ionization mode, at the facilities of the Institute of Biomedical Chemistry of RAMS. Sample solution (200 mg/L) was injected into the ESI source using a microliter pump at 90 μL/h flow-rate, pressure of nebulizer gas of 138 kPa and pressure of the drying gas of 103 kPa. A source heater temperature of 200°C was maintained to ensure rapid dissolution in ionized droplets. The spectra were acquired within a time domain of 4 megawords and 300 scans were accumulated for each spectrum. Resolving power was 530 000 at m/z = 400. First, mass-spectrum was externally calibrated using synthetic carboxyl-containing polystyrene standard, as described in [86], and internal calibration was systematically performed against residual peaks of fatty acids [87]. Molecular identifications were performed with the proprietary “Transhumus” software, based on the total mass difference statistics algorithm, designed by A. Grigoriev [88]. The generated CHO formulas were validated by the following constraints (O/C ratio ≤ 1, H/C ratio ≤ 2, element counts (C ≤ 120, H ≤ 200, O ≤ 60) and mass accuracy window < 1 ppm).

### *In vitro* study

The study was performed by Eurofins Cerep (France) using standardized Diversity Profile - P9 panel, comprising 53 receptors, 14 ion channels, 5 transporters and 27 enzymes (results from enzyme assays are not disclosed in this article). Binding of BP-Cx-1 to receptors, ion channels and transporters was examined with radioligand binding assays. Assay parameters are presented in the Supplementary File 4.

Binding results are expressed as a percentage inhibition of control specific binding obtained in the presence of BP-Cx-1:

100–((measured specific binding/control specific binding) × 100%)

Specific binding values correspond to values obtained in the presence of BP-Cx-1, less than nonspecific (background) signal. Inhibition effects greater than 50% were considered significant.

### ChEMBL data mining

Data processing was carried out using Python 2.7 scripts. Database management was carried out either in InstantJChem 17.2.6.0 [89] or in DataWarrior 4.6.1. [90].

ChEMBL version 23 was used. 3748 formulae identified in FTICR mass spectrum (Supplementary File 5) were extracted as described previously [91]. In brief, all CHO formulae for molecular weight range from 200 to 800 were generated and extracted from MySQL ChEMBL, using *molecule_dictionary.full_molformula* field (Supplementary File 6). The formulae from MS were merged with all formulae extracted from ChEMBL, giving 41834 *compound_properties.molregno* primary keys (Supplementary Files 7). Fields *assays.assay_id* and *assays.description* and *target_dictionary.pref_name* were extracted using *compound_properties.molregno* as a primary key. Fields *target_dictionary.pref_name* and *assays.description* were searched with key substring list (Supplementary File 8), using a python script (Supplementary File 9), and found assays were extracted and saved to a CSV file (Supplementary File 10). For the extracted *assay_id*'s activity data and compounds were retrieved from ChEMBL, using *activities.assay_id* and *compounds_properties.molregno* as primary keys, respectively. Compounds were standardized using ChemAxon Standardizer v.16.8.29.0 [92] (Supplementary File 11). ChEMBL activities were standardized using a python script (Supplementary File 9) and ‘active/inactive’ (1/0) flag for all the compounds was assigned. Two types of activity fingerprints were built using a python script (Supplementary File 9) based on the flag values (Supplementary Files 2, 3). Scaffold distributions were analyzed in DataWarrior 4.6.1.

Network analysis was performed through mapping a list of targets to STRING database (http://string-db.org/) which integrates known protein-protein interactions (evidence from curated databases and experiments, minimum interaction score 0.400). GO molecular functions with highest confidence scores (*p* < 1e–06) were selected from the pathway enrichment results.

## SUPPLEMENTARY MATERIALS FIGURES AND TABLES
























