# Supplementary material for: Novel water-soluble lignin derivative BP-Cx-1: identification of components and screening of potential targets in silico and in vitro
Source: Oncotarget. 2018 Apr 6;9(26):18578–93. doi: 10.18632/oncotarget.24990 (PMC5915095; doi:10.18632/oncotarget.24990)
Supplement: Supplementary file 1 [file oncotarget-09-18578-s001.pdf]

Novel water-soluble lignin derivative BP-Cx-1: identification of components and screening of potential targets *in silico* and *in vitro*

SUPPLEMENTARY MATERIALS

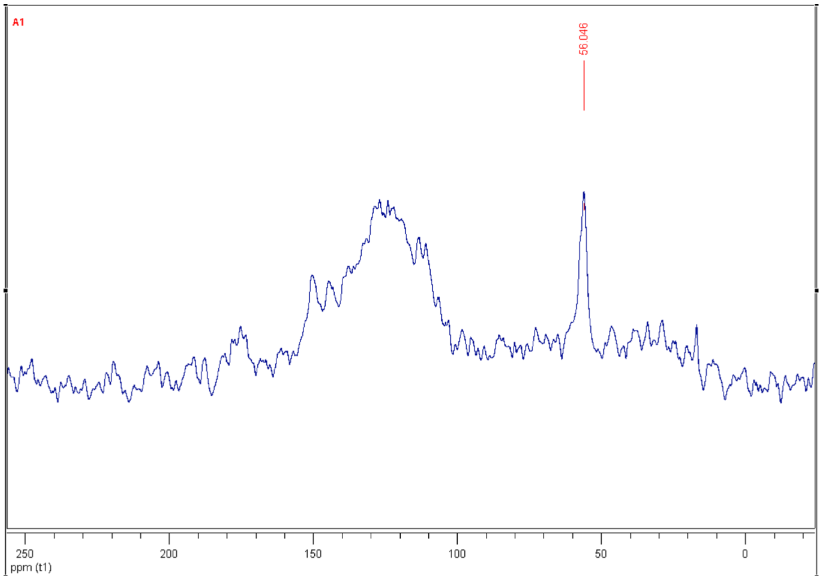

Supplementary Figure 1: ^{13}C NMR spectrum of BP-Cx-1.

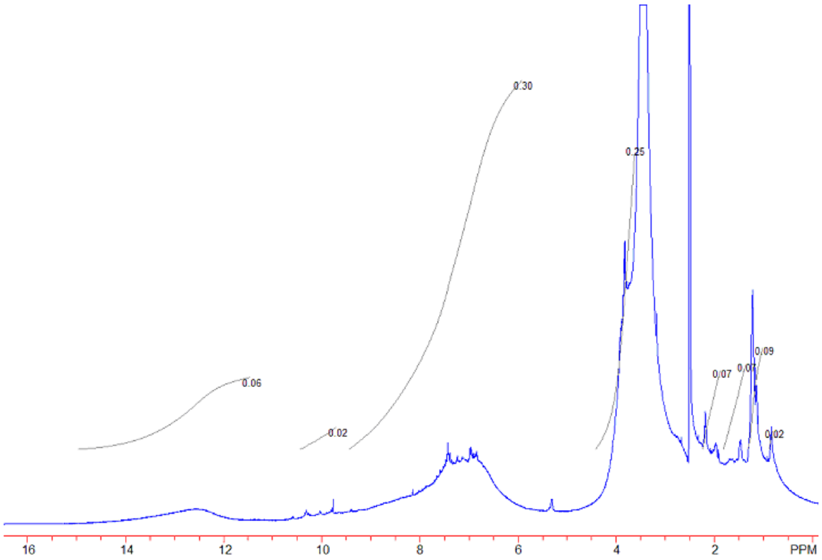

Supplementary Figure 2: ¹H NMR spectrum of BP-Cx-1 in DMSO-d_6. Signals from OCH_3 groups in 3.3–4.3 ppm region overlap with H_2O signals.

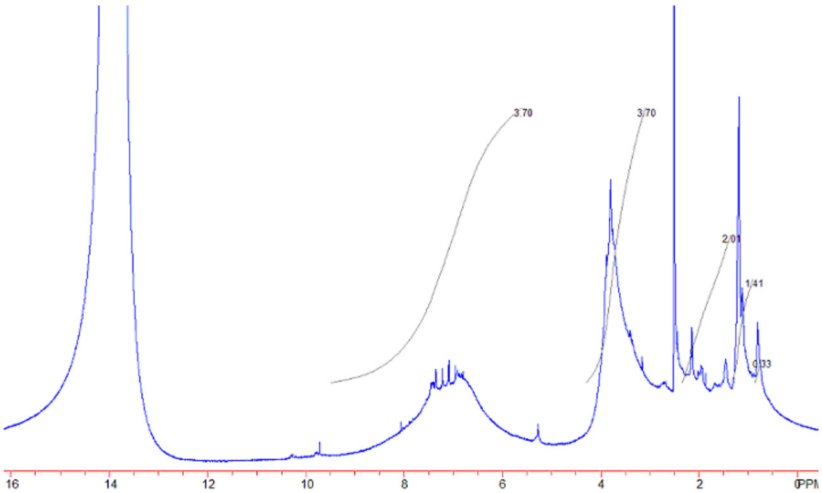

Supplementary Figure 3: ¹H NMR spectrum of BP-Cx-1 in DMSO-d_6 with addition of TFA 20%. Signals of mobile protons (H_2O, COOH, ArOH) overlap with TFA signal (13–15 ppm).

Supplementary File 1: Diversity_profile_binding_assay_results. See supplementary_File_1

Supplementary File 2: Chembl_compounds_bioactivity_profiles. See supplementary_File_2

Supplementary File 3: Fitness_of_a_chembl_compound_to_the_bp-cx-1_bioactivity_profile. See supplementary_File_3

Supplementary File 4: Diversity_profile_binding_assay_methods. See supplementary_File_4

Supplementary File 5: Formulae_identified_in_fticr_mass_spectrum. See supplementary_File_5

Supplementary File 6: Formulae_were_generated_and_extracted_from_mysql_chembl_using_molecule_dictionary.full_molformula_field. See supplementary_File_6

Supplementary File 7: Compound_properties.molregno_primary_keys. See supplementary_File_7

Supplementary File 8: Fields_target_dictionary.pref_name_and_assays.description. See supplementary_File_8

Supplementary File 9: Python_script. See supplementary_File_9

Supplementary File 10: Assays_found_by_fields_target_dictionary.pref_name_and_assays.description. See supplementary_File_10

Supplementary File 11: Chemaxon_standardizer_v.16.8.29.0. See supplementary_File_11
