# Supplementary material for: Novel water-soluble lignin derivative BP-Cx-1: identification of components and screening of potential targets in silico and in vitro
Source: Oncotarget. 2018 Apr 6;9(26):18578–93. doi: 10.18632/oncotarget.24990 (PMC5915095; doi:10.18632/oncotarget.24990)
Supplement: Supplementary file 5 [file oncotarget-09-18578-s005.docx]

*In Vitro* Pharmacology: Binding Assays

| **Assay** | **Source** | **Ligand** | **Conc.** | **Kd** | **Non Specific** | **Incubation** | **Detection Method** | **Bibl.** |
| --- | --- | --- | --- | --- | --- | --- | --- | --- |
| **Receptors** |  |  |  |  |  |  |  |  |
| **A1 *(h)***  **(antagonist** | human recombinant | [3H]DPCPX | 1 nM | 1.7 nM | DPCPX (1 µM) | 60 min RT | Scintillation counting | [1] |
| **radioligand)** | (CHO cells) |  |  |  |  |  |  |  |
| **A2A *(h)*** | human | [3H]CGS | 6 nM | 27 nM | NECA | 120 min | Scintillation | [2] |
| **(agonist** | recombinant | 21680 |  |  | (10 µM) | RT | counting |  |
| **radioligand)** | (HEK-293 |  |  |  |  |  |  |  |
|  | cells) |  |  |  |  |  |  |  |
| **A3 *(h)***  **(agonist** | human recombinant | [125I]AB-MECA | 0.15 nM | 0.22 nM | IB-MECA (1 µM) | 120 min RT | Scintillation counting | [3] |
| **radioligand)** | (HEK-293 |  |  |  |  |  |  |  |
|  | cells) |  |  |  |  |  |  |  |
| **α1 (non- selective)** | rat cerebral cortex | [3H]prazosin | 0.25 nM | 0.09 nM | prazosin (0.5 µM) | 60 min RT | Scintillation counting | [4] |
| **(antagonist** |  |  |  |  |  |  |  |  |
| **radioligand)** |  |  |  |  |  |  |  |  |
| **α2 (non- selective)** | rat cerebral cortex | [3H]RX 821002 | 0.5 nM | 0.38 nM | (-)epinephrine (100 µM) | 60 min RT | Scintillation counting | [5] |
| **(antagonist** |  |  |  |  |  |  |  |  |
| **radioligand)** |  |  |  |  |  |  |  |  |
| **β1 *(h)*** | human | [3H](-)CGP | 0.3 nM | 0.39 nM | alprenolol | 60 min | Scintillation | [6] |
| **(agonist** | recombinant | 12177 |  |  | (50 µM) | RT | counting |  |
| **radioligand)** | (HEK-293 |  |  |  |  |  |  |  |
|  | cells) |  |  |  |  |  |  |  |
| **β2 *(h)*** | human | [3H](-)CGP | 0.3 nM | 0.15 nM | alprenolol | 120 min | Scintillation | [7] |
| **(agonist** | recombinant | 12177 |  |  | (50 µM) | RT | counting |  |
| **radioligand)** | (CHO cells) |  |  |  |  |  |  |  |
| **AT1 *(h)*** | human | [125I][Sar1,Ile8]- | 0.05 nM | 0.05 nM | angiotensin-II | 120 min | Scintillation | [8] |
| **(antagonist** | recombinant | AT-II |  |  | (10 µM) | 37°C | counting |  |
| **radioligand)** | (HEK-293 |  |  |  |  |  |  |  |
|  | cells) |  |  |  |  |  |  |  |
| **AT2 *(h)*** | human | [125I]CGP | 0.01 nM | 0.01 nM | angiotensin-II | 4 hr | Scintillation | [9] |
| **(agonist** | recombinant | 42112A |  |  | (1 µM) | 37°C | counting |  |
| **radioligand)** | (HEK-293 |  |  |  |  |  |  |  |
|  | cells) |  |  |  |  |  |  |  |
| **B1 *(h)*** | human | [3H]desArg10- | 0.35 nM | 0.085 nM | desArg9[Leu8]- | 60 min | Scintillation | [10] |
| **(agonist** | recombinant | KD |  |  | BK | RT | counting |  |
| **radioligand)** | (CHO cells) |  |  |  | (10 µM) |  |  |  |
| **B2 *(h)***  **(agonist** | human recombinant | [3H]bradykinin | 0.3 nM | 0.32 nM | bradykinin (1 µM) | 60 min RT | Scintillation counting | [11] |
| **radioligand)** | (CHO cells) |  |  |  |  |  |  |  |
| **CB1 *(h)***  **(agonist** | human recombinant | [3H]CP 55940 | 0.5 nM | 3.5 nM | WIN 55212-2  (10 µM) | 120 min 37°C | Scintillation counting | [12] |
| **radioligand)** | (CHO cells) |  |  |  |  |  |  |  |
| **CB2 *(h)*** | human | [3H]WIN | 0.8 nM | 1.5 nM | WIN 55212-2 | 120 min | Scintillation | [13] |
| **(agonist** | recombinant | 55212-2 |  |  | (5 µM) | 37°C | counting |  |
| **radioligand)** | (CHO cells) |  |  |  |  |  |  |  |
| **CCK1 (CCKA)**  ***(h)*** | human recombinant | [125I]CCK-8s | 0.08 nM | 0.24 nM | CCK-8s (1 µM) | 60 min RT | Scintillation counting | [14] |
| **(agonist** | (CHO cells) |  |  |  |  |  |  |  |
| **radioligand)** |  |  |  |  |  |  |  |  |

| **Assay** | **Source** | **Ligand** | **Conc.** | **Kd** | **Non Specific** | **Incubation** | **Detection Method** | **Bibl.** |
| --- | --- | --- | --- | --- | --- | --- | --- | --- |
| **CCK2 (CCKB)**  ***(h)*** | human recombinant | [125I]CCK-8s | 0.08 nM | 0.054 nM | CCK-8s (1 µM) | 60 min RT | Scintillation counting | [15] |
| **(agonist** | (CHO cells) |  |  |  |  |  |  |  |
| **radioligand)** |  |  |  |  |  |  |  |  |
| **CRF1 *(h)***  **(agonist** | human recombinant | [125I]sauvagine | 0.075 nM | 0.12 nM | sauvagine (0.5 µM) | 120 min RT | Scintillation counting | [16] |
| **radioligand)** | (CHO cells) |  |  |  |  |  |  |  |
| **D1 *(h)***  **(antagonist** | human recombinant | [3H]SCH 23390 | 0.3 nM | 0.2 nM | SCH 23390  (1 µM) | 60 min RT | Scintillation counting | [17] |
| **radioligand)** | (CHO cells) |  |  |  |  |  |  |  |
| **D2S *(h)*** | human | [3H]methyl- | 0.3 nM | 0.15 nM | (+)butaclamol | 60 min | Scintillation | [18] |
| **(antagonist** | recombinant | spiperone |  |  | (10 µM) | RT | counting |  |
| **radioligand)** | (HEK-293 |  |  |  |  |  |  |  |
|  | cells) |  |  |  |  |  |  |  |
| **D3 *(h)*** | human | [3H]methyl- | 0.3 nM | 0.085 nM | (+)butaclamol | 60 min | Scintillation | [19] |
| **(antagonist** | recombinant | spiperone |  |  | (10 µM) | RT | counting |  |
| **radioligand)** | (CHO cells) |  |  |  |  |  |  |  |
| **D4.4 *(h)*** | human | [3H]methyl- | 0.3 nM | 0.19 nM | (+)butaclamol | 60 min | Scintillation | [20] |
| **(antagonist** | recombinant | spiperone |  |  | (10 µM) | RT | counting |  |
| **radioligand)** | (CHO cells) |  |  |  |  |  |  |  |
| **ETA *(h)*** | human | [125I]endothelin | 0.03 nM | 0.03 nM | endothelin-1 | 120 min | Scintillation | [21] |
| **(agonist** | recombinant | -1 |  |  | (100 nM) | 37°C | counting |  |
| **radioligand)** | (CHO cells) |  |  |  |  |  |  |  |
| **ETB *(h)*** | human | [125I]endothelin | 0.03 nM | 0.04 nM | endothelin-1 | 120 min | Scintillation | [22] |
| **(agonist** | recombinant | -1 |  |  | (0.1 µM) | 37°C | counting |  |
| **radioligand)** | (CHO cells) |  |  |  |  |  |  |  |
| **GABA** | rat cerebral | [3H]GABA | 10 nM | 15 nM | GABA | 60 min | Scintillation | [23] |
| **(non-** | cortex |  |  |  | (100 µM) | RT | counting |  |
| **selective)** |  |  |  |  |  |  |  |  |
| **(agonist** |  |  |  |  |  |  |  |  |
| **radioligand)** |  |  |  |  |  |  |  |  |
| **H1 *(h)***  **(antagonist** | human recombinant | [3H]pyrilamine | 1 nM | 1.7 nM | pyrilamine (1 µM) | 60 min RT | Scintillation counting | [24] |
| **radioligand)** | (HEK-293 |  |  |  |  |  |  |  |
|  | cells) |  |  |  |  |  |  |  |
| **H2 *(h)***  **(antagonist** | human recombinant | [125I]APT | 0.075 nM | 2.9 nM | tiotidine (100 µM) | 120 min RT | Scintillation counting | [25] |
| **radioligand)** | (CHO cells) |  |  |  |  |  |  |  |
| **H3 *(h)*** | human | [3H]Nα-Me- | 1 nM | 0.32 nM | (R)α-Me- | 60 min | Scintillation | [26] |
| **(agonist** | recombinant | histamine |  |  | histamine | RT | counting |  |
| **radioligand)** | (CHO cells) |  |  |  | (1 µM) |  |  |  |
| **I2** | rat cerebral | [3H]idazoxan | 2 nM | 4 nM | cirazoline | 30 min | Scintillation | [27] |
| **(antagonist** | cortex | (+ 1 µM |  |  | (10 µM) | RT | counting |  |
| **radioligand)** |  | yohimbine) |  |  |  |  |  |  |
| **BLT1 (LTB4)**  ***(h)*** | human recombinant | [3H]LTB4 | 0.2 nM | 0.2 nM | LTB4 (0.2 µM) | 60 min RT | Scintillation counting | [28] |
| **(agonist** | (CHO cells) |  |  |  |  |  |  |  |
| **radioligand)** |  |  |  |  |  |  |  |  |
| **CysLT1 (LTD4)**  ***(h)*** | human recombinant | [3H]LTD4 | 0.3 nM | 0.24 nM | LTD4 (1 µM) | 60 min RT | Scintillation counting | [29] |
| **(agonist** | (CHO cells) |  |  |  |  |  |  |  |
| **radioligand)** |  |  |  |  |  |  |  |  |
| **MC4 *(h)*** | human | [125I]NDP-α- | 0.05 nM | 0.54 nM | NDP-α-MSH | 120 min | Scintillation | [30] |
| **(agonist** | recombinant | MSH |  |  | (1 µM) | 37°C | counting |  |
| **radioligand)** | (CHO cells) |  |  |  |  |  |  |  |
| **MT1 (ML1A) *(h)*** | human | [125I]2- | 0.01 nM | 0.04 nM | melatonin | 60 min | Scintillation | [31] |
| **(agonist** | recombinant | iodomelatonin |  |  | (1 µM) | RT | counting |  |
| **radioligand)** | (CHO cells) |  |  |  |  |  |  |  |
| **M (non-** | rat cerebral | [3H]QNB | 0.05 nM | 0.01 nM | atropine | 120 min | Scintillation | [32] |
| **selective)** | cortex |  |  |  | (1 µM) | RT | counting |  |
| **(antagonist** |  |  |  |  |  |  |  |  |
| **radioligand)** |  |  |  |  |  |  |  |  |

| **Assay** | **Source** | **Ligand** | **Conc.** | **Kd** | **Non Specific** | **Incubation** | **Detection Method** | **Bibl.** |
| --- | --- | --- | --- | --- | --- | --- | --- | --- |
| **NK1 *(h)*** | U373MG | [125I]- | 0.05 nM | 0.04 nM | [Sar9,Met(O2)1 | 30 min | Scintillation | [33] |
| **(agonist** | uppsala | Substance P |  |  | 1]-SP | RT | counting |  |
| **radioligand)** |  | LYS3 |  |  | (1 µM) |  |  |  |
| **NK2 *(h)*** | human | [125I]NKA | 0.1 nM | 0.12 nM | [Nleu10]-NKA | 60 min | Scintillation | [34] |
| **(agonist** | recombinant |  |  |  | (4-10) | RT | counting |  |
| **radioligand)** | (CHO cells) |  |  |  | (300 nM) |  |  |  |
| **NK3 *(h)*** | human | [3H]SR 142801 | 0.4 nM | 0.47 nM | SB 222200 | 120 min | Scintillation | [35] |
| **(antagonist** | recombinant |  |  |  | (10 µM) | RT | counting |  |
| **radioligand)** | (CHO cells) |  |  |  |  |  |  |  |
| **Y (non-** | rat cerebral | [125I]peptide | 0.05 nM | 0.1 nM | NPY | 120 min | Scintillation | [36] |
| **selective)** | cortex | YY |  |  | (1 µM) | RT | counting |  |
| **(agonist** |  |  |  |  |  |  |  |  |
| **radioligand)** |  |  |  |  |  |  |  |  |
| **N neuronal** | SH-SY5Y cells | [3H]cytisine | 0.6 nM | 0.3 nM | nicotine | 120 min | Scintillation | [37] |
| **α4β2 *(h)*** | (human |  |  |  | (10 µM) | 4°C | counting |  |
| **(agonist** | recombinant) |  |  |  |  |  |  |  |
| **radioligand)** |  |  |  |  |  |  |  |  |
| **opioid** | rat cerebral | [3H]naloxone | 1 nM | 2.6 nM | naloxone | 40 min | Scintillation | [38] |
| **(non-** | cortex |  |  |  | (1 µM) | RT | counting |  |
| **selective)** |  |  |  |  |  |  |  |  |
| **(antagonist** |  |  |  |  |  |  |  |  |
| **radioligand)** |  |  |  |  |  |  |  |  |
| **NOP (ORL1)** | human | [3H]nociceptin | 0.2 nM | 0.4 nM | nociceptin | 60 min | Scintillation | [39] |
| ***(h)*** | recombinant |  |  |  | (1 µM) | RT | counting |  |
| **(agonist** | (HEK-293 |  |  |  |  |  |  |  |
| **radioligand)** | cells) |  |  |  |  |  |  |  |
| **PPARγ *(h)*** | human | [3H]rosiglitazon | 5 nM | 5.7 nM | rosiglitazone | 120 min | Scintillation | [40] |
| **(agonist** | recombinant | e |  |  | (10 µM) | 4°C | counting |  |
| **radioligand)** | (*E. coli*) |  |  |  |  |  |  |  |
| **EP2 *(h)*** | human | [3H]PGE2 | 3 nM | 3 nM | PGE2 | 120 min | Scintillation | [41] |
| **(agonist** | recombinant |  |  |  | (10 µM) | RT | counting |  |
| **radioligand)** | (HEK-293 |  |  |  |  |  |  |  |
|  | cells) |  |  |  |  |  |  |  |
| **IP (PGI2) *(h)*** | human | [3H]iloprost | 6 nM | 8 nM | iloprost | 60 min | Scintillation | [41] |
| **(agonist** | recombinant |  |  |  | (10 µM) | RT | counting |  |
| **radioligand)** | (HEK-293 |  |  |  |  |  |  |  |
|  | cells) |  |  |  |  |  |  |  |
| **P2Y** | rat cerebral | [35S]dATPαS | 10 nM | 10 nM | dATPαS | 60 min | Scintillation | [42] |
| **(agonist** | cortex |  |  |  |  | RT | counting |  |
| **radioligand)** |  |  |  |  |  |  |  |  |
| **Serotonin (5-** | rat cerebral | [3H] Serotonin | 2 nM | 0.61 nM | Serotonin (5- | 10 min | Scintillation | [43] |
| **Hydroxytrypta** | cortex | (5-HT) |  |  | HT)(10.0 µM) | 37°C | counting |  |
| **mine) 5-HT1,** |  |  |  |  |  |  |  |  |
| **Non-Selective** |  |  |  |  |  |  |  |  |
| **sigma (non-** | Jurkat cells | [3H]DTG | 10 nM | 41 nM | Haloperidol (10 | 120 min | Scintillation | [44] |
| **selective) (h)** | (endogenous) |  |  |  | µM) | RT | counting |  |
| **(agonist** |  |  |  |  |  |  |  |  |
| **radioligand)** |  |  |  |  |  |  |  |  |
| **GR *(h)*** | IM-9 cells | [3H]dexametha | 1.5 nM | 1.5 nM | triamcinolone | 6 hr | Scintillation | [45] |
| **(agonist** | (cytosol) | sone |  |  | (10 µM) | 4°C | counting |  |
| **radioligand)** |  |  |  |  |  |  |  |  |
| **ER (non-** | MCF-7 cells | [3H]estradiol | 0.4 nM | 0.2 nM | 17-β-estradiol | 20 hr | Scintillation | [46] |
| **selective) *(h)*** | (cytosol) |  |  |  | (6 µM) | 4°C | counting |  |
| **(agonist** |  |  |  |  |  |  |  |  |
| **radioligand)** |  |  |  |  |  |  |  |  |
| **PR *(h)*** | T47D cells | [3H]progestero | 0.5 nM | 2 nM | promegestone | 20 hr | Scintillation | [47] |
| **(agonist** | (cytosol) | ne |  |  | (1 µM) | 4°C | counting |  |
| **radioligand)** |  |  |  |  |  |  |  |  |
| **AR *(h)*** | LNCaP cells | [3H]methyltrien | 1 nM | 0.8 nM | mibolerone | 24 hr | Scintillation | [48] |
| **(agonist** | (cytosol) | olone |  |  | (1 µM) | 4°C | counting |  |
| **radioligand)** |  |  |  |  |  |  |  |  |
| **TRH1 *(h)*** | human | [3H]Me-TRH | 2 nM | 3.9 nM | TRH | 120 min | Scintillation | [49] |
| **(agonist** | recombinant |  |  |  | (10 µM) | 4°C | counting |  |
| **radioligand)** | (CHO cells) |  |  |  |  |  |  |  |

| **Assay** | **Source** | **Ligand** | **Conc.** | **Kd** | **Non Specific** | **Incubation** | **Detection Method** | **Bibl.** |
| --- | --- | --- | --- | --- | --- | --- | --- | --- |
| **V1 a *(h)***  **(agonist** | human recombinant | [3H]AVP | 0.3 nM | 0.5 nM | AVP  (1 µM) | 60 min RT | Scintillation counting | [50] |
| **radioligand)** | (CHO cells) |  |  |  |  |  |  |  |
| **V2 *(h)***  **(agonist** | human recombinant | [3H]AVP | 0.3 nM | 0.76 nM | AVP  (1 µM) | 120 min RT | Scintillation counting | [50] |
| **radioligand)** | (CHO cells) |  |  |  |  |  |  |  |
| **Ion channels** |  |  |  |  |  |  |  |  |
| **BZD (central)** | rat cerebral | [3H]flunitrazepa | 0.4 nM | 2.1 nM | diazepam | 60 min | Scintillation | [51] |
| **(agonist** | cortex | m |  |  | (3 µM) | 4°C | counting |  |
| **radioligand)** |  |  |  |  |  |  |  |  |
| **AMPA** | rat cerebral | [3H]AMPA | 8 nM | 82 nM | L-glutamate | 60 min | Scintillation | [52] |
| **(agonist** | cortex |  |  |  | (1 mM) | 4°C | counting |  |
| **radioligand)** |  |  |  |  |  |  |  |  |
| **kainate** | rat cerebral | [3H]kainic acid | 5 nM | 19 nM | L-glutamate | 60 min | Scintillation | [53] |
| **(agonist** | cortex |  |  |  | (1 mM) | 4°C | counting |  |
| **radioligand)** |  |  |  |  |  |  |  |  |
| **NMDA** | rat cerebral | [3H]CGP | 5 nM | 23 nM | L-glutamate | 60 min | Scintillation | [54] |
| **(antagonist** | cortex | 39653 |  |  | (100 µM) | 4°C | counting |  |
| **radioligand)** |  |  |  |  |  |  |  |  |
| **PCP** | rat cerebral | [3H]TCP | 10 nM | 13 nM | MK 801 | 120 min | Scintillation | [55] |
| **(antagonist** | cortex |  |  |  | (10 µM) | 37°C | counting |  |
| **radioligand)** |  |  |  |  |  |  |  |  |
| **P2X** | rat urinary | [3H]α,β-MeATP | 3 nM | 2.6 nM | α,β-MeATP | 120 min | Scintillation | [56] |
| **(agonist** | bladder |  |  |  | (10 µM) | 4°C | counting |  |
| **radioligand)** |  |  |  |  |  |  |  |  |
| **Ca2+ channel** | rat cerebral | [3H]nitrendipine | 0.1 nM | 0.18 nM | nitrendipine | 90 min | Scintillation | [57] |
| **(L,** | cortex |  |  |  | (1 µM) | RT | counting |  |
| **dihydropyridi** |  |  |  |  |  |  |  |  |
| **ne site)** |  |  |  |  |  |  |  |  |
| **(antagonist** |  |  |  |  |  |  |  |  |
| **radioligand)** |  |  |  |  |  |  |  |  |
| **Ca2+ channel** | rat cerebral | [3H]diltiazem | 15 nM | 52 nM | diltiazem | 120 min | Scintillation | [58] |
| **(L, diltiazem** | cortex |  |  |  | (10 µM) | RT | counting |  |
| **site)** |  |  |  |  |  |  |  |  |
| **(benzothiazep** |  |  |  |  |  |  |  |  |
| **ines)** |  |  |  |  |  |  |  |  |
| **(antagonist** |  |  |  |  |  |  |  |  |
| **radioligand)** |  |  |  |  |  |  |  |  |
| **Ca2+ channel** | rat cerebral | [3H]D888 | 3 nM | 3 nM | D 600 | 120 min | Scintillation | [59] |
| **(L, verapamil** | cortex |  |  |  | (10 µM) | RT | counting |  |
| **site)** |  |  |  |  |  |  |  |  |
| **(phenylalkyla** |  |  |  |  |  |  |  |  |
| **mine)** |  |  |  |  |  |  |  |  |
| **(antagonist** |  |  |  |  |  |  |  |  |
| **radioligand)** |  |  |  |  |  |  |  |  |
| **KATP channel** | rat cerebral | [3H]glibenclami | 0.1 nM | 0.05 nM | glibenclamide | 60 min | Scintillation | [60] |
| **(antagonist** | cortex | de |  |  | (1 µM) | RT | counting |  |
| **radioligand)** |  |  |  |  |  |  |  |  |
| **KV channel** | rat cerebral | [125I]α- | 0.01 nM | 0.04 nM | α-dendrotoxin | 60 min | Scintillation | [61] |
| **(antagonist** | cortex | dendrotoxin |  |  | (50 nM) | RT | counting |  |
| **radioligand)** |  |  |  |  |  |  |  |  |
| **SKCa channel (antagonist** | rat cerebral cortex | [125I]apamin | 0.007 nM | 0.007 nM | apamin (100 nM) | 60 min 4°C | Scintillation counting | [62] |
| **radioligand)** |  |  |  |  |  |  |  |  |
| **Na+ channel** | rat cerebral | [3H]batrachoto | 10 nM | 91 nM | veratridine | 60 min | Scintillation | [63] |
| **(site 2)** | cortex | xinin |  |  | (300 µM) | 37°C | counting |  |
| **(antagonist** |  |  |  |  |  |  |  |  |
| **radioligand)** |  |  |  |  |  |  |  |  |
| **Cl- channel** | rat cerebral | [35S]TBPS | 3 nM | 14.6 nM | picrotoxinin | 120 min | Scintillation | [64] |
| **(GABA-gated)** | cortex |  |  |  | (20 µM) | RT | counting |  |
| **(antagonist** |  |  |  |  |  |  |  |  |
| **radioligand)** |  |  |  |  |  |  |  |  |

| **Assay** | **Source** | **Ligand** | **Conc.** | **Kd** | **Non Specific** | **Incubation** | **Detection Method** | **Bibl.** |
| --- | --- | --- | --- | --- | --- | --- | --- | --- |
| **Transporters** |  |  |  |  |  |  |  |  |
| **norepinephrine** | human | [3H]nisoxetine | 1 nM | 2.9 nM | desipramine | 120 min | Scintillation | [65] |
| **transporter *(h)*** | recombinant |  |  |  | (1 µM) | 4°C | counting |  |
| **(antagonist** | (CHO cells) |  |  |  |  |  |  |  |
| **radioligand)** |  |  |  |  |  |  |  |  |
|  |  |  |  |  |  |  |  |  |
| **dopamine** | human | [3H]BTCP | 4 nM | 4.5 nM | BTCP | 120 min | Scintillation | [66] |
| **transporter *(h)*** | recombinant |  |  |  | (10 µM) | 4°C | counting |  |
| **(antagonist** | (CHO cells) |  |  |  |  |  |  |  |
| **radioligand)** |  |  |  |  |  |  |  |  |
| **GABA** | rat cerebral | [3H]GABA | 10 nM | 4600 nM | GABA | 30 min | Scintillation | [67] |
| **transporter** | cortex | (+ 10 µM |  |  | (1 mM) | RT | counting |  |
| **(antagonist** |  | isoguvacine) |  |  |  |  |  |  |
| **radioligand)** |  | (+ 10 µM |  |  |  |  |  |  |
|  |  | baclofen) |  |  |  |  |  |  |
| **choline** | human | [3H]hemicholini | 3 nM | 3.9 nM | hemicholinium- | 60 min | Scintillation | [68] |
| **transporter** | recombinant | um-3 |  |  | 3 | RT | counting |  |
| **(CHT1) *(h)*** | (CHO cells) |  |  |  | (10 µM) |  |  |  |
| **(antagonist** |  |  |  |  |  |  |  |  |
| **radioligand)** |  |  |  |  |  |  |  |  |
| **5-HT** | human | [3H]imipramine | 2 nM | 1.7 nM | imipramine | 60 min | Scintillation | [69] |
| **transporter** | recombinant |  |  |  | (10 µM) | RT | counting |  |
| ***(h)*** | (CHO cells) |  |  |  |  |  |  |  |
| **(antagonist** |  |  |  |  |  |  |  |  |
| **radioligand)** |  |  |  |  |  |  |  |  |

Bibliography

1. Townsend-Nicholson, A.; Schofield, P. R. A threonine residue in the seventh transmembrane domain of the human A1 adenosine receptor mediates specific agonist binding. *J. Biol. Chem.* **1994**, *269*, 2373–2376.

2. Luthin, D. R.; Olsson, R. A.; Thompson, R. D.; Sawmiller, D. R.; Linden, J. Characterization of two affinity states of adenosine A2a receptors with a new radioligand, 2-[2-(4-amino-3-[125I]iodophenyl)ethylamino]adenosine. *Mol. Pharmacol.* **1995**, *47*, 307–313.

3. Salvatore, C. A.; Jacobson, M. A.; Taylor, H. E.; Linden, J.; Johnson, R. G. Molecular cloning and characterization of the human A3 adenosine receptor. *Proc. Natl. Acad. Sci. U. S. A.* **1993**, *90*, 10365–10369.

4. Greengrass, P.; Bremner, R. Binding characteristics of 3H-prazosin to rat brain alpha-adrenergic receptors. *Eur. J. Pharmacol.* **1979**, *55*, 323–326.

5. Uhlén, S.; Wikberg, J. E. Rat spinal cord alpha 2-adrenoceptors are of the alpha 2A-subtype: comparison with alpha 2A- and alpha 2B-adrenoceptors in rat spleen, cerebral cortex and kidney using 3H-RX821002 ligand binding. *Pharmacol. Toxicol.* **1991**, *69*, 341–350.

6. Levin, M. C.; Marullo, S.; Muntaner, O.; Andersson, B.; Magnusson, Y. The myocardium-protective Gly-49 variant of the beta 1-adrenergic receptor exhibits constitutive activity and increased desensitization and down-regulation. *J. Biol. Chem.* **2002**, *277*, 30429–30435, doi:10.1074/jbc.M200681200.

7. Joseph, S. S.; Lynham, J. A.; Colledge, W. H.; Kaumann, A. J. Binding of (-)-[3H]-CGP12177 at two sites in recombinant human beta 1-adrenoceptors and interaction with beta-blockers. *Naunyn. Schmiedebergs Arch. Pharmacol.* **2004**, *369*, 525–532, doi:10.1007/s00210-004-0884-y.

8. Le, M. T.; De Backer, J.-P.; Hunyady, L.; Vanderheyden, P. M. L.; Vauquelin, G. Ligand binding and functional properties of human angiotensin AT1 receptors in transiently and stably expressed CHO-K1 cells. *Eur. J. Pharmacol.* **2005**, *513*, 35–45, doi:10.1016/j.ejphar.2005.02.029.

9. Tsuzuki, S.; Ichiki, T.; Nakakubo, H.; Kitami, Y.; Guo, D. F.; Shirai, H.; Inagami, T. Molecular cloning and expression of the gene encoding human angiotensin II type 2 receptor. *Biochem. Biophys. Res. Commun.* **1994**, *200*, 1449–1454, doi:10.1006/bbrc.1994.1613.

10. Jones, C.; Phillips, E.; Davis, C.; Arbuckle, J.; Yaqoob, M.; Burgess, G. M.; Docherty, R. J.; Webb, M.; Bevan, S. J.; McIntyre, P. Molecular characterisation of cloned bradykinin B1 receptors from rat and human. *Eur. J. Pharmacol.* **1999**, *374*, 423–433.

11. Pruneau, D.; Luccarini, J. M.; Fouchet, C.; Defrêne, E.; Franck, R. M.; Loillier, B.; Duclos, H.; Robert, C.; Cremers, B.; Bélichard, P.; Paquet, J. L. LF 16.0335, a novel potent and selective nonpeptide antagonist of the human bradykinin B2 receptor. *Br. J. Pharmacol.* **1998**, *125*, 365–372, doi:10.1038/sj.bjp.0702083.

12. Rinaldi-Carmona, M.; Calandra, B.; Shire, D.; Bouaboula, M.; Oustric, D.; Barth, F.; Casellas, P.; Ferrara, P.; Le Fur, G. Characterization of two cloned human CB1 cannabinoid receptor isoforms. *J. Pharmacol. Exp. Ther.* **1996**, *278*, 871–878.

13. Munro, S.; Thomas, K. L.; Abu-Shaar, M. Molecular characterization of a peripheral receptor for cannabinoids. *Nature* **1993**, *365*, 61–65, doi:10.1038/365061a0.

14. Bignon, E.; Bachy, A.; Boigegrain, R.; Brodin, R.; Cottineau, M.; Gully, D.; Herbert, J. M.; Keane, P.; Labie, C.; Molimard, J. C.; Olliero, D.; Oury-Donat, F.; Petereau, C.; Prabonnaud, V.; Rockstroh, M. P.; Schaeffer, P.; Servant, O.; Thurneyssen, O.; Soubrié, P.; Pascal, M.; Maffrand, J. P.; Le Fur, G. SR146131: a new potent, orally active, and selective nonpeptide cholecystokinin subtype 1 receptor agonist. I. In vitro studies. *J. Pharmacol. Exp. Ther.* **1999**, *289*, 742–751.

15. Lee, Y. M.; Beinborn, M.; McBride, E. W.; Lu, M.; Kolakowski, L. F.; Kopin, A. S. The human brain cholecystokinin-B/gastrin receptor. Cloning and characterization. *J. Biol. Chem.* **1993**, *268*, 8164–8169.

16. Palchaudhuri, M. R.; Wille, S.; Mevenkamp, G.; Spiess, J.; Fuchs, E.; Dautzenberg, F. M. Corticotropin-releasing factor receptor type 1 from Tupaia belangeri--cloning, functional expression and tissue distribution. *Eur. J. Biochem.* **1998**, *258*, 78–84.

17. Zhou, Q. Y.; Grandy, D. K.; Thambi, L.; Kushner, J. A.; Van Tol, H. H.; Cone, R.; Pribnow, D.; Salon, J.; Bunzow, J. R.; Civelli, O. Cloning and expression of human and rat D1 dopamine receptors. *Nature* **1990**, *347*, 76–80, doi:10.1038/347076a0.

18. Grandy, D. K.; Marchionni, M. A.; Makam, H.; Stofko, R. E.; Alfano, M.; Frothingham, L.; Fischer, J. B.; Burke-Howie, K. J.; Bunzow, J. R.; Server, A. C. Cloning of the cDNA and gene for a human D2 dopamine receptor. *Proc. Natl. Acad. Sci. U. S. A.* **1989**, *86*, 9762–9766.

19. MacKenzie, R. G.; VanLeeuwen, D.; Pugsley, T. A.; Shih, Y. H.; Demattos, S.; Tang, L.; Todd, R. D.; O’Malley, K. L. Characterization of the human dopamine D3 receptor expressed in transfected cell lines. *Eur. J. Pharmacol.* **1994**, *266*, 79–85.

20. Tol, H. H. M. V.; Wu, C. M.; Guan, H.-C.; Ohara, K.; Bunzow, J. R.; Civelli, O.; Kennedy, J.; Seeman, P.; Niznik, H. B.; Jovanovic, V. Multiple dopamine D4 receptor variants in the human population. *Nature* **1992**, *358*, 149.

21. Buchan, K. W.; Alldus, C.; Christodoulou, C.; Clark, K. L.; Dykes, C. W.; Sumner, M. J.; Wallace, D. M.; White, D. G.; Watts, I. S. Characterization of three non-peptide endothelin receptor ligands using human cloned ETA and ETB receptors. *Br. J. Pharmacol.* **1994**, *112*, 1251–1257.

22. Fuchs, S.; Amiel, J.; Claudel, S.; Lyonnet, S.; Corvol, P.; Pinet, F. Functional characterization of three mutations of the endothelin B receptor gene in patients with Hirschsprung’s disease: evidence for selective loss of Gi coupling. *Mol. Med. Camb. Mass* **2001**, *7*, 115–124.

23. Tsuji, A.; Sato, H.; Kume, Y.; Tamai, I.; Okezaki, E.; Nagata, O.; Kato, H. Inhibitory effects of quinolone antibacterial agents on gamma-aminobutyric acid binding to receptor sites in rat brain membranes. *Antimicrob. Agents Chemother.* **1988**, *32*, 190–194.

24. Smit, M. J.; Timmerman, H.; Hijzelendoorn, J. C.; Fukui, H.; Leurs, R. Regulation of the human histamine H1 receptor stably expressed in Chinese hamster ovary cells. *Br. J. Pharmacol.* **1996**, *117*, 1071–1080.

25. Leurs, R.; Smit, M. J.; Menge, W. M.; Timmerman, H. Pharmacological characterization of the human histamine H2 receptor stably expressed in Chinese hamster ovary cells. *Br. J. Pharmacol.* **1994**, *112*, 847–854.

26. Lovenberg, T. W.; Roland, B. L.; Wilson, S. J.; Jiang, X.; Pyati, J.; Huvar, A.; Jackson, M. R.; Erlander, M. G. Cloning and functional expression of the human histamine H3 receptor. *Mol. Pharmacol.* **1999**, *55*, 1101–1107.

27. Brown, C. M.; MacKinnon, A. C.; McGrath, J. C.; Spedding, M.; Kilpatrick, A. T. Alpha 2-adrenoceptor subtypes and imidazoline-like binding sites in the rat brain. *Br. J. Pharmacol.* **1990**, *99*, 803–809.

28. Yokomizo, T.; Kato, K.; Hagiya, H.; Izumi, T.; Shimizu, T. Hydroxyeicosanoids bind to and activate the low affinity leukotriene B4 receptor, BLT2. *J. Biol. Chem.* **2001**, *276*, 12454–12459, doi:10.1074/jbc.M011361200.

29. Martin, V.; Sawyer, N.; Stocco, R.; Unett, D.; Lerner, M. R.; Abramovitz, M.; Funk, C. D. Molecular cloning and functional characterization of murine cysteinyl-leukotriene 1 (CysLT(1)) receptors. *Biochem. Pharmacol.* **2001**, *62*, 1193–1200.

30. Schiöth, H. B.; Muceniece, R.; Wikberg, J. E. Characterization of the binding of MSH-B, HB-228, GHRP-6 and 153N-6 to the human melanocortin receptor subtypes. *Neuropeptides* **1997**, *31*, 565–571.

31. Witt-Enderby, P. A.; Dubocovich, M. L. Characterization and regulation of the human ML1A melatonin receptor stably expressed in Chinese hamster ovary cells. *Mol. Pharmacol.* **1996**, *50*, 166–174.

32. Richards, M. H. Rat hippocampal muscarinic autoreceptors are similar to the M2 (cardiac) subtype: comparison with hippocampal M1, atrial M2 and ileal M3 receptors. *Br. J. Pharmacol.* **1990**, *99*, 753–761.

33. Heuillet, E.; Ménager, J.; Fardin, V.; Flamand, O.; Bock, M.; Garret, C.; Crespo, A.; Fallourd, A. M.; Doble, A. Characterization of a human NK1 tachykinin receptor in the astrocytoma cell line U 373 MG. *J. Neurochem.* **1993**, *60*, 868–876.

34. Aharony, D.; Little, J.; Powell, S.; Hopkins, B.; Bundell, K. R.; McPheat, W. L.; Gordon, R. D.; Hassall, G.; Hockney, R.; Griffin, R. Pharmacological characterization of cloned human NK-2 (neurokinin A) receptor expressed in a baculovirus/Sf-21 insect cell system. *Mol. Pharmacol.* **1993**, *44*, 356–363.

35. Sarau, H. M.; Griswold, D. E.; Potts, W.; Foley, J. J.; Schmidt, D. B.; Webb, E. F.; Martin, L. D.; Brawner, M. E.; Elshourbagy, N. A.; Medhurst, A. D.; Giardina, G. A.; Hay, D. W. Nonpeptide tachykinin receptor antagonists: I. Pharmacological and pharmacokinetic characterization of SB 223412, a novel, potent and selective neurokinin-3 receptor antagonist. *J. Pharmacol. Exp. Ther.* **1997**, *281*, 1303–1311.

36. Goldstein, M.; Kusano, N.; Adler, C.; Meller, E. Characterization of central neuropeptide Y receptor binding sites and possible interactions with alpha 2-adrenoceptors. *Prog. Brain Res.* **1986**, *68*, 331–335.

37. Gopalakrishnan, M.; Monteggia, L. M.; Anderson, D. J.; Molinari, E. J.; Piattoni-Kaplan, M.; Donnelly-Roberts, D.; Arneric, S. P.; Sullivan, J. P. Stable expression, pharmacologic properties and regulation of the human neuronal nicotinic acetylcholine alpha 4 beta 2 receptor. *J. Pharmacol. Exp. Ther.* **1996**, *276*, 289–297.

38. Childers, S. R.; Creese, I.; Snowman, A. M.; Synder, S. H. Opiate receptor binding affected differentially by opiates and opioid peptides. *Eur. J. Pharmacol.* **1979**, *55*, 11–18.

39. Ardati, A.; Henningsen, R. A.; Higelin, J.; Reinscheid, R. K.; Civelli, O.; Monsma, F. J. Interaction of [3H]orphanin FQ and 125I-Tyr14-orphanin FQ with the orphanin FQ receptor: kinetics and modulation by cations and guanine nucleotides. *Mol. Pharmacol.* **1997**, *51*, 816–824.

40. Ferry, G.; Bruneau, V.; Beauverger, P.; Goussard, M.; Rodriguez, M.; Lamamy, V.; Dromaint, S.; Canet, E.; Galizzi, J. P.; Boutin, J. A. Binding of prostaglandins to human PPARgamma: tool assessment and new natural ligands. *Eur. J. Pharmacol.* **2001**, *417*, 77–89.

41. Abramovitz, M.; Adam, M.; Boie, Y.; Carrière, M.; Denis, D.; Godbout, C.; Lamontagne, S.; Rochette, C.; Sawyer, N.; Tremblay, N. M.; Belley, M.; Gallant, M.; Dufresne, C.; Gareau, Y.; Ruel, R.; Juteau, H.; Labelle, M.; Ouimet, N.; Metters, K. M. The utilization of recombinant prostanoid receptors to determine the affinities and selectivities of prostaglandins and related analogs. *Biochim. Biophys. Acta* **2000**, *1483*, 285–293.

42. Simon, J.; Webb, T. E.; Barnard, E. A. Characterization of a P2Y purinoceptor in the brain. *Pharmacol. Toxicol.* **1995**, *76*, 302–307.

43. Middlemiss, D. N. Stereoselective blockade at [3H]5-HT binding sites and at the 5-HT autoreceptor by propranolol. *Eur. J. Pharmacol.* **1984**, *101*, 289–293.

44. Ganapathy, M. E.; Prasad, P. D.; Huang, W.; Seth, P.; Leibach, F. H.; Ganapathy, V. Molecular and ligand-binding characterization of the sigma-receptor in the Jurkat human T lymphocyte cell line. *J. Pharmacol. Exp. Ther.* **1999**, *289*, 251–260.

45. Clark, A. F.; Lane, D.; Wilson, K.; Miggans, S. T.; McCartney, M. D. Inhibition of dexamethasone-induced cytoskeletal changes in cultured human trabecular meshwork cells by tetrahydrocortisol. *Invest. Ophthalmol. Vis. Sci.* **1996**, *37*, 805–813.

46. Kurata, Y.; Tabata, Y.; Shinei, R.; Iizuka, Y.; Masuda, N. T.; Kurihara, K.; Okonogi, T.; Hoshiko, S. Endocrinological properties of two novel nonsteroidal progesterone receptor modulators, CP8816 and CP8863. *J. Pharmacol. Exp. Ther.* **2005**, *313*, 916–920, doi:10.1124/jpet.104.074146.

47. Sarup, J. C.; Rao, K. V.; Williams, R. E.; Fox, C. F. Resolution of high and low affinity progesterone receptors from human breast carcinoma T47D cells. *J. Biol. Chem.* **1988**, *263*, 5624–5633.

48. Zava, D. T.; Landrum, B.; Horwitz, K. B.; McGuire, W. L. Androgen receptor assay with [3H]methyltrienolone (R1881) in the presence of progesterone receptors. *Endocrinology* **1979**, *104*, 1007–1012, doi:10.1210/endo-104-4-1007.

49. Hinuma, S.; Hosoya, M.; Ogi, K.; Tanaka, H.; Nagai, Y.; Onda, H. Molecular cloning and functional expression of a human thyrotropin-releasing hormone (TRH) receptor gene. *Biochim. Biophys. Acta* **1994**, *1219*, 251–259.

50. Tahara, A.; Saito, M.; Sugimoto, T.; Tomura, Y.; Wada, K.; Kusayama, T.; Tsukada, J.; Ishii, N.; Yatsu, T.; Uchida, W.; Tanaka, A. Pharmacological characterization of the human vasopressin receptor subtypes stably expressed in Chinese hamster ovary cells. *Br. J. Pharmacol.* **1998**, *125*, 1463–1470, doi:10.1038/sj.bjp.0702220.

51. Speth, R. C.; Wastek, G. J.; Yamamura, H. I. Benzodiazepine receptors: Temperature dependence of [3H]flunitrazepam binding. *Life Sci.* **1979**, *24*, 351–357, doi:10.1016/0024-3205(79)90331-X.

52. Murphy, D. E.; Snowhill, E. W.; Williams, M. Characterization of quisqualate recognition sites in rat brain tissue using DL-[3H]alpha-amino-3-hydroxy-5-methylisoxazole-4-propionic acid (AMPA) and a filtration assay. *Neurochem. Res.* **1987**, *12*, 775–781.

53. Monaghan, D. T.; Cotman, C. W. The distribution of [3H]kainic acid binding sites in rat CNS as determined by autoradiography. *Brain Res.* **1982**, *252*, 91–100.

54. Sills, M. A.; Fagg, G.; Pozza, M.; Angst, C.; Brundish, D. E.; Hurt, S. D.; Wilusz, E. J.; Williams, M. [3H]CGP 39653: a new N-methyl-D-aspartate antagonist radioligand with low nanomolar affinity in rat brain. *Eur. J. Pharmacol.* **1991**, *192*, 19–24.

55. Vignon, J.; Privat, A.; Chaudieu, I.; Thierry, A.; Kamenka, J. M.; Chicheportiche, R. [3H]thienyl-phencyclidine ([3H]TCP) binds to two different sites in rat brain. Localization by autoradiographic and biochemical techniques. *Brain Res.* **1986**, *378*, 133–141.

56. Bo, X. N.; Burnstock, G. High- and low-affinity binding sites for [3H]-alpha, beta-methylene ATP in rat urinary bladder membranes. *Br. J. Pharmacol.* **1990**, *101*, 291–296.

57. Gould, R. J.; Murphy, K. M.; Snyder, S. H. [3H]nitrendipine-labeled calcium channels discriminate inorganic calcium agonists and antagonists. *Proc. Natl. Acad. Sci. U. S. A.* **1982**, *79*, 3656–3660.

58. Schoemaker, H.; Langer, S. Z. [3H]diltiazem binding to calcium channel antagonists recognition sites in rat cerebral cortex. *Eur. J. Pharmacol.* **1985**, *111*, 273–277.

59. Reynolds, I. J.; Snowman, A. M.; Snyder, S. H. (-)-[3H] desmethoxyverapamil labels multiple calcium channel modulator receptors in brain and skeletal muscle membranes: differentiation by temperature and dihydropyridines. *J. Pharmacol. Exp. Ther.* **1986**, *237*, 731–738.

60. Angel, I.; Bidet, S. The binding site for [3H]glibenclamide in the rat cerebral cortex does not recognize K-channel agonists or antagonists other than sulphonylureas. *Fundam. Clin. Pharmacol.* **1991**, *5*, 107–115.

61. Sorensen, R. G.; Blaustein, M. P. Rat brain dendrotoxin receptors associated with voltage-gated potassium channels: dendrotoxin binding and receptor solubilization. *Mol. Pharmacol.* **1989**, *36*, 689–698.

62. Hugues, M.; Duval, D.; Kitabgi, P.; Lazdunski, M.; Vincent, J. P. Preparation of a pure monoiodo derivative of the bee venom neurotoxin apamin and its binding properties to rat brain synaptosomes. *J. Biol. Chem.* **1982**, *257*, 2762–2769.

63. Brown, G. B. 3H-batrachotoxinin-A benzoate binding to voltage-sensitive sodium channels: inhibition by the channel blockers tetrodotoxin and saxitoxin. *J. Neurosci. Off. J. Soc. Neurosci.* **1986**, *6*, 2064–2070.

64. Lewin, A. H.; de Costa, B. R.; Rice, K. C.; Skolnick, P. meta- and para-isothiocyanato-t-butylbicycloorthobenzoate: irreversible ligands of the gamma-aminobutyric acid-regulated chloride ionophore. *Mol. Pharmacol.* **1989**, *35*, 189–194.

65. Pacholczyk, T.; Blakely, R. D.; Amara, S. G. Expression cloning of a cocaine- and antidepressant-sensitive human noradrenaline transporter. *Nature* **1991**, *350*, 350–354, doi:10.1038/350350a0.

66. Pristupa, Z. B.; Wilson, J. M.; Hoffman, B. J.; Kish, S. J.; Niznik, H. B. Pharmacological heterogeneity of the cloned and native human dopamine transporter: disassociation of [3H]WIN 35,428 and [3H]GBR 12,935 binding. *Mol. Pharmacol.* **1994**, *45*, 125–135.

67. Shank, R. P.; Baldy, W. J.; Mattucci, L. C.; Villani, F. J. Ion and temperature effects on the binding of gamma-aminobutyrate to its receptors and the high-affinity transport system. *J. Neurochem.* **1990**, *54*, 2007–2015.

68. Apparsundaram, S.; Ferguson, S. M.; George, A. L.; Blakely, R. D. Molecular cloning of a human, hemicholinium-3-sensitive choline transporter. *Biochem. Biophys. Res. Commun.* **2000**, *276*, 862–867, doi:10.1006/bbrc.2000.3561.

69. Tatsumi, M.; Jansen, K.; Blakely, R. D.; Richelson, E. Pharmacological profile of neuroleptics at human monoamine transporters. *Eur. J. Pharmacol.* **1999**, *368*, 277–283.
