# Supplementary material for: Novel water-soluble lignin derivative BP-Cx-1: identification of components and screening of potential targets in silico and in vitro
Source: Oncotarget. 2018 Apr 6;9(26):18578–93. doi: 10.18632/oncotarget.24990 (PMC5915095; doi:10.18632/oncotarget.24990)
Supplement: Supplementary file 10 [file oncotarget-09-18578-s010.docx]

"""

Created on Sat Oct 7 19:03:09 2017

@author: alex

"""

import pandas as pd

import numpy as np

import re

fey=pd.read_csv('/home/alex/HS_project/Molecules/Fedoros-formulas.csv', sep=';')

fey['Formula']='C'+fey['C'].astype(str)+'H'+fey['H'].astype(str)+'O'+fey['O'].astype(str)

per=pd.read_csv('/home/alex/HS_project/ChemMedChem/Supplementary_FIles/S4.csv')

feyper=pd.merge(fey, per, on='Formula', how='inner')

feyper=feyper[['Formula','molregno']]

feyper.to_csv('/home/alex/HS_project/Molecules/Supplementary_fin/S3.csv', index=None)

pref_wo=pd.read_csv('/home/alex/HS_project/Molecules/database.csv', sep=',')

typ=pd.read_csv('/home/alex/HS_project/Molecules/assays_types.csv', sep=',')

pref=pd.merge(pref_wo, typ, on='assay_id', how='inner')

pref['dupl']=pref['assay_id'].astype(str)+pref['pref_name']

pref=pref.drop_duplicates(['dupl'])

pref=pref[['assay_id', 'description', 'pref_name']]

dict_rec=pd.read_csv('/home/alex/HS_project/Molecules/Supplementary_fin/hs_search_substrings.csv')

list_filter=['Thyrotropin-releasing hormone receptor 2',

'Adenosine A2b receptor',

'Beta-3 adrenergic receptor',

'Dopamine D5 receptor',

'Vitamin D',

'Metabotropic glutamate receptor'

'serotonin(.*)5.HT[0,2,3,4,5,6,7,8,9](.*)receptor',

'Prostanoid EP1 receptor',

'Prostanoid EP3 receptor',

'Prostanoid EP4 receptor',

'Prostanoid FP receptor',

'Prostanoid DP receptor',

'type',

'Prostaglandin D2 receptor',

'PGD-2',

'sigma'

]

for item in list_filter:

pref['pref_name']=pref['pref_name'].str.replace(str(item), '', case=False)

pref=pref[~pref['description'].isnull()]

#pref=pref[pref['assay_type']=='B']

pref=pref[pref['description'].str.contains('displacement|affinity|binding', case=False)==True]

pref=pref[pref['description'].str.contains('agonist activity|antagonist activity', case=False)==False]

pref=pref[pref['description'].str.contains('retinoic(.*)acid(.*)beta(.*)receptor', case=False)==False]

dflist=[]

count=0

#pref['description']=pref['description'].str.contains('displacement|affinity|binding', case=False)

for item, row in dict_rec.iterrows():

#row='\A(.*)(\W*)endothelin(.*)receptor(.*)A'

#print row['Name']

#if(row['Name']=='\A(.*)(\W*)endothelin(.*)receptor(.*)A' ):

# print row['Name']

# mask=pref[pref['pref_name'].str.contains(str(row['Name']), case=False)==True]

# print mask

mask=pref[pref['pref_name'].str.contains(str(row['Name']), case=False)==True]

slength=len(mask)

if slength!=0:

Name=[str(row['Name'])]*slength

Target=[str(row['Target'])]*slength

hs_activity=[str(row['hs_activity'])]*slength

mask['Name']=pd.Series(Name, index=mask.index)

mask['Target']=pd.Series(Target, index=mask.index)

mask['hs_activity']=pd.Series(hs_activity, index=mask.index)

count+=1

print count

dflist.append(mask)

res1=pd.concat(dflist)

dflist=[]

dflist_bad=[]

resbad1=pd.DataFrame()

error1=0

error2=0

for assay in res1['assay_id'].unique().tolist():

mask=res1[res1['assay_id']==assay]

if(len(mask)==1):

dflist.append(mask)

else:

mask['dupl']=mask['pref_name']+mask['hs_activity'].astype(str)

mask=mask.drop_duplicates(['dupl'])

del mask['dupl']

if(len(mask[mask['hs_activity']!='ND']['hs_activity'].unique())==1):

mask=mask[mask['hs_activity']!='ND']

dflist.append(mask)

elif(len(mask[mask['hs_activity']!='ND'])==0):

mask=mask.drop_duplicates(['hs_activity'])

dflist.append(mask)

else:

error1+=1

print error1

dflist_bad.append(mask)

res1=pd.concat(dflist)

if(len(dflist_bad)>0):

resbad1=pd.concat(dflist_bad)

#res1_nd=res1[res1['hs_activity']=='ND']

#res1=res1[res1['hs_activity']!='ND']

pref=pref[~pref['assay_id'].isin(res1['assay_id'].tolist())]

#dflist=[pref, res1]

#pref=pd.concat(dflist)

dflist=[]

list_filter2=['alpha2c',

'alpha1d',

'beta3',

'EP1receptor',

'EP3receptor',

'EP4receptor',

'FPreceptor',

'DPreceptor',

'RARbeta2',

'prostoglandind2',

'ThromboxaneA2',

'TXA2PGH2',

'TRH2',

'3HPG2',

'gangliosideGM1',

]

for item in list_filter:

pref=pref[pref['description'].str.contains(str(item), case=False)==False]

pref['description_cut']=pref['description'].str.replace('[^a-zA-Z0-9]', '', case=False)

for item in list_filter2:

pref=pref[pref['description_cut'].str.contains(str(item), case=False)==False]

pref['description_partlycut']=pref['description'].str.replace('[^a-zA-Z]', ' ', case=False)

pref['description_partlycut']=pref['description_partlycut'].str.replace('\s+', ' ', case=False)

#pref[(pref['pref_name']=='Unchecked')&(pref['description_cut'].str.contains('opioidreceptor', case=False)==True)].to_csv('/home/alex/test.csv')

dict_rec['Name_cut']=dict_rec['Name'].str.replace('\\\A', '', case=False)

dict_rec['Name_cut']=dict_rec['Name_cut'].str.replace('\\\W', '', case=False)

dict_rec['Name_cut']=dict_rec['Name_cut'].str.replace('\\\s', '', case=False)

dict_rec['Name_cut']=dict_rec['Name_cut'].str.replace('\\\B', '', case=False)

dict_rec['Name_cut']=dict_rec['Name_cut'].str.replace('[^a-zA-Z0-9]', '', case=False)

#dict_rec['Name_cut']=dict_rec['Name'].str.replace('\(\.\*\)', '', case=False)

#dict_rec['Name_cut']=dict_rec['Name_cut'].str.replace('\\\A', '', case=False)

#dict_rec['Name_cut']=dict_rec['Name_cut'].str.replace('\\\W', '', case=False)

#dict_rec['Name_cut']=dict_rec['Name_cut'].str.replace('\(\*\)', '', case=False)

#dict_rec['Name_cut']=dict_rec['Name_cut'].str.replace('W', '', case=True)

for item, row in dict_rec.iterrows():

#print row['Name_cut']

try:

re.search('[0-9]', str(row['Name_cut']))

mask=pref[pref['description_cut'].str.contains(str(row['Name_cut']), case=False)==True]

except:

mask=pref[pref['description_partlycut'].str.contains(str(row['Name_cut']), case=False)==True]

#print mask

slength=len(mask)

if slength!=0:

Name=[str(row['Name'])]*slength

Target=[str(row['Target'])]*slength

hs_activity=[str(row['hs_activity'])]*slength

mask['Name']=pd.Series(Name, index=mask.index)

mask['Target']=pd.Series(Target, index=mask.index)

mask['hs_activity']=pd.Series(hs_activity, index=mask.index)

count+=1

print count

dflist.append(mask)

res2=pd.concat(dflist)

del res2['description_cut']

dflist=[]

dflist_bad=[]

error2=0

for assay in res2['assay_id'].unique().tolist():

mask=res2[res2['assay_id']==assay]

if(len(mask)==1):

dflist.append(mask)

else:

mask['dupl']=mask['pref_name']+mask['hs_activity'].astype(str)

mask=mask.drop_duplicates(['dupl'])

del mask['dupl']

if(len(mask[mask['hs_activity']!='ND']['hs_activity'].unique())==1):

mask=mask[mask['hs_activity']!='ND']

dflist.append(mask)

elif(len(mask[mask['hs_activity']!='ND'])==0):

mask=mask.drop_duplicates(['hs_activity'])

dflist.append(mask)

else:

error2+=1

print error2

dflist_bad.append(mask)

res2=pd.concat(dflist)

if(len(dflist_bad)>0):

resbad2=pd.concat(dflist_bad)

#res2=res2[~res2['assay_id'].isin(res1['assay_id'].tolist())]

#res2=res2.drop_duplicates(['assay_id'])

#res2.to_csv('/home/alex/test.csv')

dflist=[res1, res2]

res=pd.concat(dflist)

dflistn=[]

dfbad=[]

count=0

for assay in res['assay_id'].tolist():

mask=res[res['assay_id']==assay]

#print mask

if(len(mask)>1):

mask=mask[mask['hs_activity']!='ND']

mask['dupl']=mask['Target']+mask['hs_activity'].astype(str)

mask=mask.drop_duplicates(['dupl'])

del mask['dupl']

if(len(mask)>1):

if((len(mask[mask['Target'].str.contains('bradykinin', case=False)==True])>0)&(len(mask[mask['Target'].str.contains('cannabinoid', case=False)==True])>0)):

mask=mask[mask['Target'].str.contains('bradykinin', case=False)==False]

mask['dupl']=mask['Target']+mask['hs_activity'].astype(str)

mask=mask.drop_duplicates(['dupl'])

del mask['dupl']

#mask=mask.drop_duplicates(['hs_activity'])

#print mask

elif((len(mask[mask['Target'].str.contains('opioid', case=False)==True])>0)&(len(mask[mask['Target'].str.contains('sigma', case=False)==True])>0)):

mask=mask[mask['Target'].str.contains('opioid', case=False)==False]

mask['dupl']=mask['Target']+mask['hs_activity'].astype(str)

mask=mask.drop_duplicates(['dupl'])

del mask['dupl']

elif(len(mask['Target'].unique().tolist())>1):

print 'error'

count+=1

print count

print mask

dfbad.append(mask)

else:

mask=mask.drop_duplicates(['Target'])

#print mask

dflistn.append(mask)

else:

dflistn.append(mask)

resfin=pd.concat(dflistn)

resfin['dupl']=resfin['assay_id'].astype(str)+resfin['Target']+resfin['hs_activity'].astype(str)

resfin=resfin.drop_duplicates(['dupl'])

del resfin['dupl']

#resfin=resfin[resfin['hs_activity']!='ND']

resfin=resfin[['assay_id', 'description', 'pref_name']]

resfin.to_csv('/home/alex/HS_project/Molecules/Supplementary_fin/S4_new.csv')

#####################checking

old=pd.read_csv('/home/alex/HS_project/Molecules/assays_fed.csv')

old=old[old['hs_activity']!='ND']

old=old.drop_duplicates(['assay_id'])

old=old[old['description_x'].str.contains('agonist activity|antagonist activity', case=False)==False]

old[~old['assay_id'].isin(resfin['assay_id'].unique().tolist())].to_csv('/home/alex/HS_project/Molecules/check_again.csv')

act=pd.read_csv('/home/alex/S4_new.csv')

act=act[act['molregno'].isin(feyper['molregno'].unique().tolist())]

stru=act[['molregno', 'canonical_smiles']]

stru=stru.drop_duplicates(['molregno'])

stru.to_csv('/home/alex/HS_project/Molecules/Supplementary_Files/structures_nonfiltered.csv')

stru=pd.read_csv('/home/alex/HS_project/Molecules/Supplementary_Files/structures_clusters.csv')

stract=pd.merge(stru, act, on='molregno', how='inner')

stacre=pd.merge(stract, resfin, on='assay_id', how='inner')

#stacre.columns

stacre['dupl']=stacre['Target']+stacre['Cluster_number'].astype(str)

stacre.drop_duplicates(['dupl'])

fin=pd.read_csv('/home/alex/HS_project/Molecules/fin.csv')

#####################################activity standartisation##########################

fin['act_flag']=np.nan

dflist=[]

##########activity_comment

def activity_standart(row):

if np.isnan(row['act_flag']):

if(row['standard_type']=='RBA'):

if((row['standard_units']=='%')&((row['standard_value']<50))):

row['act_flag']=0

elif((row['standard_units']=='%')&((row['standard_value']>=50))):

row['act_flag']=1

elif((row['standard_units']=='nM')&((row['standard_relation']=='<')|(row['standard_relation']=='='))&(row['standard_value']<10000)):

row['act_flag']=1

elif((row['standard_units']=='uM')&((row['standard_relation']=='<')|(row['standard_relation']=='='))&(row['standard_value']<10)):

row['act_flag']=1

elif((row['standard_units']=='%')&((row['standard_relation']=='<')|(row['standard_relation']=='='))&(row['standard_value']<=50)):

row['act_flag']=1

elif(((row['standard_units']=='nM')|(row['standard_units']=='uM')|(row['standard_units']=='%')|(row['standard_units']=='ug.mL-1'))&(row['standard_relation']=='>')):

row['act_flag']=0

elif(((row['standard_units']=='nM')|(row['standard_units']=='uM')|(row['standard_units']=='%')|(row['standard_units']=='ug.mL-1'))&(row['standard_relation']=='>=')):

row['act_flag']=0

elif(((row['standard_units']=='nM'))&(row['standard_relation']=='=')&(row['standard_value']>=10000)):

row['act_flag']=0

elif(((row['standard_units']=='uM'))&(row['standard_relation']=='=')&(row['standard_value']>=10)):

row['act_flag']=0

elif((row['standard_units']=='%')&(row['standard_relation']=='=')&(row['standard_value']>50)):

row['act_flag']=0

elif(row['standard_units']=='ug.mL-1'):

row['standard_value']=row['standard_value']/1000/row['Mol Weight']

row['standard_units']='uM'

if((row['standard_units']=='uM')&((row['standard_relation']=='<')|(row['standard_relation']=='='))&(row['standard_value']<10)):

row['act_flag']=1

elif(((row['standard_units']=='uM'))&(row['standard_relation']=='=')&(row['standard_value']>=10)):

row['act_flag']=0

elif(row['standard_type']=='pKi(uM)'):

# print row['standard_value']

row['standard_value']=10**((-1)*(row['standard_value']))

row['standard_units']='uM'

dflist.append(row)

if((row['standard_units']=='uM')&((row['standard_relation']=='<')|(row['standard_relation']=='='))&(row['standard_value']<10)):

row['act_flag']=1

elif(((row['standard_units']=='uM'))&(row['standard_relation']=='=')&(row['standard_value']>=10)):

row['act_flag']=0

return row

else:

return row

fin.loc[fin['activity_comment'].str.contains('Not Active', case=False)==True, 'act_flag']=0

fin.loc[fin['activity_comment'].str.contains('Active', case=False)==True, 'act_flag']=1

#fin[fin['standard_units']=='ug.mL-1']['stadard_value']=fin[fin['standard_units']=='ug.mL-1']['standard_value']/1000

#fin.loc[fin['standard_units']=='ug.mL-1', 'standard_units']='uM'

fin=fin.apply(activity_standart, axis=1)

fin['stdstr_id']=fin['Cluster_number']

fin['dupl']

finres=fin[fin['act_flag'].isnull()==False]

finres

fey=finres[['stdstr_id', 'assay_id', 'standard_value', 'standard_units', 'standard_type', 'standard_relation', 'description', 'pref_name', 'Name', 'Target', 'act_flag', 'hs_activity', 'Structure']]

fey.to_csv('/home/alex/HS_project/Molecules/finres_12_11_17.csv', index=None)

fin[fin['act_flag'].isnull()==True].to_csv('/home/alex/bad_types.csv')

fey=pd.read_csv('/home/alex/HS_project/Molecules/finres_12_11_17.csv')

fingerprint=pd.DataFrame(index=fey['stdstr_id'].unique().tolist(), columns=dict_rec['Target'].unique().tolist())

fingerprint['stdstr_id']=fingerprint.index

dflist=[]

def fingerprint_stat(group):

#print group

temp=group.groupby(['Target', 'stdstr_id', 'hs_activity'])['act_flag'].mean().reset_index()

if(len(temp[~temp['act_flag'].isin([0,1])])>0):

dflist.append(temp[~temp['act_flag'].isin([0,1])])

temp.loc[temp['act_flag']<0.5, 'act_flag']=0

temp.loc[temp['act_flag']>=0.5, 'act_flag']=1

temp['act_fin']=np.nan

#temp.loc[temp['act_flag']==temp['hs_activity'], 'act_fin']=1

#temp.loc[temp['act_flag']!=temp['hs_activity'], 'act_fin']=-1

for column in group['Target'].unique().tolist():

# print type(column)

fingerprint.loc[fingerprint['stdstr_id'].isin(temp['stdstr_id'].unique().tolist()), column]=int(temp.loc[temp['Target']==column, 'act_flag'])

return group

grouped=fey.groupby(['stdstr_id'])

k=grouped.apply(fingerprint_stat)

del fingerprint['stdstr_id']

fingerprint['fit_score']=fingerprint.sum(axis=1)

#ideal=fey[['Target', 'hs_activity']]

fingerprint['stdstr_id']=fingerprint.index

form=fey[['stdstr_id', 'Structure']]

form=form.drop_duplicates(['stdstr_id'])

finres=pd.merge(fingerprint, form, on='stdstr_id', how='inner')

finres.to_csv('/home/alex/HS_project/fingerprints_v2.csv', index=None)

act[~act['molregno'].isin(stru['molregno'].tolist())]

#==============================================================================

# put all the activities and Nan values to fingerprint

#then compare all fingerprints

#==============================================================================

resbad=pd.concat(dfbad)

old=pd.read_csv('/home/alex/HS_project/Molecules/S5.csv')

res['check']=np.nan

res.loc[res['assay_id'].isin(old['assay_id'].tolist()), 'check']='old'

res=res.fillna('new')

res=res.drop_duplicates(subset=['assay_id'])

res.to_csv('/home/alex/HS_project/Molecules/Supplementary_Files/S2.csv', index=None)

##################################ligand selection###################################

desc=pd.read_csv('/home/alex/HS_project/Molecules/Supplementary_Files/S2.csv')

desc=resfin

#desc=desc[['assay_id', 'description']]

desc=desc.drop_duplicates(subset=['assay_id'])

ligname=pd.read_csv('/home/alex/HS_project/Molecules/ligand_names.csv')

ligname['ligand_name_search']=ligname['ligand_name'].str.replace('\W','', case=False)

desc['description_search']=desc['description'].str.replace('\W','', case=False)

dflist=[]

count=0

for item, row in ligname.iterrows():

mask=desc[desc['description_search'].str.contains(row['ligand_name_search'], case=False)==True]

slength=len(mask)

if slength!=0:

Name=[str(row['ligand_name'])]*slength

search=[str(row['ligand_name_search'])]*slength

prefn=[str(row['pref_name_new'])]*slength

kd=[str(row['hs_kd_nm'])]*slength

pname=[str(row['panel_name'])]*slength

hsact=[str(row['hs_activity'])]*slength

mask['ligand_name']=pd.Series(Name, index=mask.index)

mask['ligand_name_search']=pd.Series(search, index=mask.index)

mask['hs_kd_nm']=pd.Series(kd, index=mask.index)

mask['panel_name']=pd.Series(pname, index=mask.index)

mask['hs_activity']=pd.Series(hsact, index=mask.index)

mask['pref_name_new']=pd.Series(prefn, index=mask.index)

count+=1

print count

dflist.append(mask)

reslig=pd.concat(dflist)

del reslig['description_search']

del reslig['ligand_name_search']

del reslig['pref_name_new']

res_nolig=res[~res['assay_id'].isin(reslig['assay_id'].tolist())]

res_nolig['ligand_name']='ND'

res_nolig['hs_kd_nm']='ND'

res_nolig['panel_name']='ND'

#res_nolig['hs_activity']=np.nan

dflist=[reslig, res_nolig]

fin=pd.concat(dflist)

fin.to_csv('/home/alex/HS_project/Molecules/Supplementary_Files/assays.csv')

#reslig=reslig[~(reslig['pref_name_new']=='ND')]

reslig.to_csv('/home/alex/HS_project/Molecules/Supplementary_Files/S3_all.csv')

####################################################################################

ass=pd.read_csv('/home/alex/assays_1.csv')

reslig=pd.read_csv('/home/alex/HS_project/Molecules/Supplementary_Files/S2.csv')

reslig=reslig.iloc[:,1:]

#reslig['name']=reslig['assay_id'].astype(str)+reslig['pref_name_new']

reslig=res

reslig=reslig.drop_duplicates(['assay_id'])

#ass=ass[ass['assay_id'].isin(reslig['assay_id'].tolist())]

#reslig=reslig[['assay_id', 'hs_kd_nm']]

resass=pd.merge(reslig, ass, on='assay_id', how='inner')

stru=pd.read_csv('/home/alex/HS_project/Molecules/Supplementary_Files/structures_stdstr_id.csv')

resass['molregno']=resass['molregno_y']

resfin=pd.merge(resass, stru, on='molregno', how='left')

resfin.to_csv('/home/alex/HS_project/Molecules/assays.csv', index=None)

lik=pd.read_csv('/home/alex/assays_old.csv')

lik.loc[(lik['assay_id'].isin(resfin['assay_id'].tolist())), 'old']=1

lik=lik.fillna(0)

lik=lik[lik['old']==0]

lik.to_csv('/home/alex/test_1.csv')

reslig.to_csv('/home/alex/HS_project/Molecules/Supplementary_Files/S3_all.csv')

old=pd.read_csv('/home/alex/HS_project/Molecules/S5.csv')

resfin['check']=np.nan

resfin.loc[resfin['assay_id'].isin(old['assay_id'].tolist()), 'check']='old'

resfin=resfin.fillna('new')

resfin.to_csv('/home/alex/HS_project/Molecules/S6_all.csv')

####################################################################################

for item in ligname['ligand_name_search'].tolist():

dflist.append()

#ligname['ligand_name_search']=ligname['ligand_name_search'].str.replace('\W','', case=False)

#################################

res=pd.read_csv('/home/alex/HS_project/Molecules/Supplementary_Files/S2.csv')

feyper=pd.read_csv('/home/alex/HS_project/Molecules/Supplementary_Files/S1.csv')

resform=pd.merge(feyper, res, on='molregno', how='inner')

structures=pd.read_csv('/home/alex/HS_project/Molecules/Supplementary_Files/structures_stdstr_id.csv', sep=',')

resform=resform[['description', 'pref_name', 'molregno', 'assay_id', 'Formula']]

str_pref=pd.merge(structures, resform, on='molregno', how='inner')

str_pref['name']=str_pref['stdstr_id'].astype(str)+str_pref['pref_name']+str_pref['assay_id'].astype(str)+str_pref['Formula']

str_pref=str_pref.drop_duplicates(['name'])

str_pref.to_csv('/home/alex/HS_project/Molecules/Supplementary_Files/structures_targets_assays.csv', index=None)
