# Supplementary material for: Novel water-soluble lignin derivative BP-Cx-1: identification of components and screening of potential targets in silico and in vitro
Source: Oncotarget. 2018 Apr 6;9(26):18578–93. doi: 10.18632/oncotarget.24990 (PMC5915095; doi:10.18632/oncotarget.24990)
Supplement: Supplementary file 12 [file oncotarget-09-18578-s012.doc]

"<?xml version=""1.0"" encoding=""UTF-8""?>" "<StandardizerConfiguration Version=""0.1"">"
 <Actions> " <StripSalts ID=""Strip Salts"" dontremovelastcomponent=""true"" usedefaultsalts=""true""/>" " <DisconnectMetalAtoms ID=""Disconnect Metal Atoms""/>" " <RemoveSolvents ID=""Remove Solvents"" usedefaultsolvents=""true""/>" " <RemoveFragment ID=""Remove Fragment"" Measure=""atomCount"" Method=""keepLargest""/>" " <Aromatize ID=""Aromatize"" Type=""general""/>" " <Dearomatize ID=""Dearomatize""/>" " <Tautomerize ID=""Tautomerize""/>"
 </Actions> </StandardizerConfiguration> 
